# Salivary gland branching morphogenesis: a quantitative systems analysis of the Eda/Edar/NFκB paradigm

**DOI:** 10.1186/1471-213X-9-32

**Published:** 2009-06-06

**Authors:** Michael Melnick, Robert D Phair, Smadar A Lapidot, Tina Jaskoll

**Affiliations:** 1 Laboratory for Developmental Genetics, USC, Los Angeles, CA, USA; 2 Integrative Bioinformatics Inc, Los Altos, CA, USA

## Abstract

**Background:**

Ectodysplasin-A appears to be a critical component of branching morphogenesis. Mutations in mouse *Eda *or human *EDA *are associated with absent or hypoplastic sweat glands, sebaceous glands, lacrimal glands, salivary glands (SMGs), mammary glands and/or nipples, and mucous glands of the bronchial, esophageal and colonic mucosa. In this study, we utilized *Eda*^*Ta *^(Tabby) mutant mice to investigate how a marked reduction in functional Eda propagates with time through a defined genetic subcircuit and to test the proposition that canonical NFκB signaling is sufficient to account for the differential expression of developmentally regulated genes in the context of *Eda *polymorphism.

**Results:**

The quantitative systems analyses do not support the stated hypothesis. For most NFκB-regulated genes, the observed time course of gene expression is nearly unchanged in Tabby (*Eda*^*Ta*^) as compared to wildtype mice, as is NFκB itself. Importantly, a subset of genes is dramatically differentially expressed in Tabby (*Edar*, *Fgf8*, *Shh*, *Egf*, *Tgfa*, *Egfr*), strongly suggesting the existence of an alternative Eda-mediated transcriptional pathway pivotal for SMG ontogeny. Experimental and *in silico *investigations have identified C/EBPα as a promising candidate.

**Conclusion:**

In Tabby SMGs, upregulation of the Egf/Tgfα/Egfr pathway appears to mitigate the potentially severe abnormal phenotype predicted by the downregulation of Fgf8 and Shh. Others have suggested that the buffering of the phenotypic outcome that is coincident with variant Eda signaling could be a common mechanism that permits viable and diverse phenotypes, normal and abnormal. Our results support this proposition. Further, if branching epithelia use variations of a canonical developmental program, our results are likely applicable to understanding the phenotypes of other branching organs affected by *Eda *(*EDA*) mutation.

## Background

Branching morphogenesis is a common mechanism of mammalian development (salivary glands, lungs, mammary glands, pancreas, kidney, etc.), and has been a classic topic of study for generations of developmental biologists [1]. Based on recent findings regarding signal transduction pathways and transcriptional control, it has reasonably been proposed that all branching systems use variations of a canonical developmental program [2]. Ectodysplasin-A, a protein required for epithelial differentiation, appears to be an important constituent of such a program.

Ectodysplasin-A (*Eda *in mouse, *EDA *in human) is mapped to the X-chromosome [3,4]. Eda is a glycosylated, oligomeric type II membrane protein with three collagenous repeat domains and a TNF homology domain [3-6]. Eda is shed from the cell membrane and binds as a trimer to its trimerized cognate receptor (Edar) [7,8]. Like TNF/TNFR signaling, Eda/Edar signaling is thought to be primarily through the canonical NFκB pathway [9-13]. The initial appearance of Eda and Edar proteins in *Late Pseudoglandular/Canalicular *Stage (E15) submandibular salivary glands (SMGs) *in vivo *indicate that they participate in late branching morphogenesis and histodifferentiation [14]. *In vitro *study of the Eda/Edar pathway in embryonic SMG development indicates that this pathway is important for epithelial cell proliferation, lumina formation, and histodifferentiation; exogenous Eda delivered to SMG explants upregulate NFκB activation and nuclear localization [14].

Mutations in mouse *Eda *or its human homologue *EDA *result in hypohydrotic ectodermal dysplasia (HED), a syndrome variably characterized by absent or hypoplastic teeth, hair, sweat glands, sebaceous glands, lacrimal glands, *salivary glands*, mammary glands and/or nipples, and mucous glands of the bronchial, esophageal and colonic mucosa [14-22].

The mouse *Eda*^*Ta *^(Tabby) allele is characterized by a ~2 kb deletion [3,4]. Specifically, genomic DNA hybridized with an exon 1 probe shows a deletion including the coding region, and primers for DNA flanking exon 1 fail to amplify in a PCR assay (Jackson Laboratories; ). Importantly, RT-PCR assays of embryonic *Eda*^*Ta *^(Tabby) skin reveals that *Eda*^*Ta *^transcript levels are about 10–20% of that seen in the wildtype [23,24]. This may reflect the fact that DNA sequences that regulate gene transcription occupy no fixed position relative to coding DNA regions and are often diffuse and widely dispersed, including secondary ("shadow") enhancers [25,26]. Secondary enhancers map far from the target gene and mediate activities overlapping the primary enhancer, accounting for why deletions of well-defined enhancers are sometimes associated with weak or no phenotypic abnormality [25].

In this study, we utilized *Eda*^*Ta *^(Tabby) mutant mice to investigate the *in vivo *relationship between Eda/Edar signaling and progressive (E13-NB) SMG morphogenesis and histodifferentiation. Our investigation reveals that, from the outset (E13), embryonic Tabby SMGs are smaller, exhibit fewer branches, and are developmentally delayed compared to wildtype (WT) glands. By E18, Tabby glands remain smaller with fewer presumptive acini than WT, though both display a similar degree of terminal differentiation (presumptive functional maturation), as evidenced by mucin (MucCAM/Muc10) protein expression. The key question is how altered *Eda *function is related to abnormal SMG ontogeny.

Developmental biology has progressed from studying one or two molecules at a time to studying scores of molecules. Such studies require complex experiments and mathematical analyses of their results. While the preferred approach for data analysis has been statistical, its value is limited for mechanistic understanding of signal transduction, mostly because the approach is correlational. To understand how a specific ligand/receptor binding (or lack thereof) produces a change in cell/tissue behavior, one is compelled to utilize a dynamic model of a larger relevant genetic subcircuit. Such dynamic models allow one to test different sets of unbiased assumptions and determine if the predicted behavior matches the actual.

Thus, the wider goal of the present study was to understand how a marked reduction in functional Eda propagates with time through a defined subcircuit that includes 5 signaling pathways that are both critical for SMG ontogeny and share post-activation downstream targets. More specifically, it was the central objective of our extensive *quantitative *study to demonstrate the way in which a mechanistic pathway diagram can be programmatically transformed to a corresponding system of ordinary differential equations in order to quantitatively test the proposition that the canonical NFκB signaling cascade is sufficient to account for the differential *quantitative *expression of developmentally-regulated genes in the context of *Eda *polymorphism (*Eda *v. *Eda*^*Ta*^).

Quantitative gene expression analysis and mechanistic kinetic modeling reveal that the *Eda*^*Ta *^allele is associated with significantly downregulated *Edar*, *Shh *and *Fgf8 *expression, and vastly upregulated *Egf*/*Tgfα*/*Egfr *expression. Further, the results revealed that Eda/Edar signaling is not a major determinant of NFκB signaling in normal and mutant Tabby SMG development; rather, TNF is. Moreover, the evidence strongly points to the existence of an important, alternative Eda-initiated transcriptional control pivotal to SMG development. These results are likely to be applicable to other branching organs affected by *Eda *(*EDA*) mutation.

## Results

The SMG in the *adult *Tabby mouse is smaller than the wildtype (WT) gland and is characterized by decreased granular convoluted ducts and acini; there is a significant decrease in mucin protein in the hypoplastic Tabby SMGs compared to WT, but not more than expected given the gland size differences [14,15]. To elucidate the natural history of the pathogenesis, we compared the developmental phenotypes of embryonic (E) days 13–18 Tabby and WT SMGs and relate these to multiple gene expression and putative functional integration of related pathways.

### Progressive pathogenesis of Tabby SMGs

**Figure 1:**
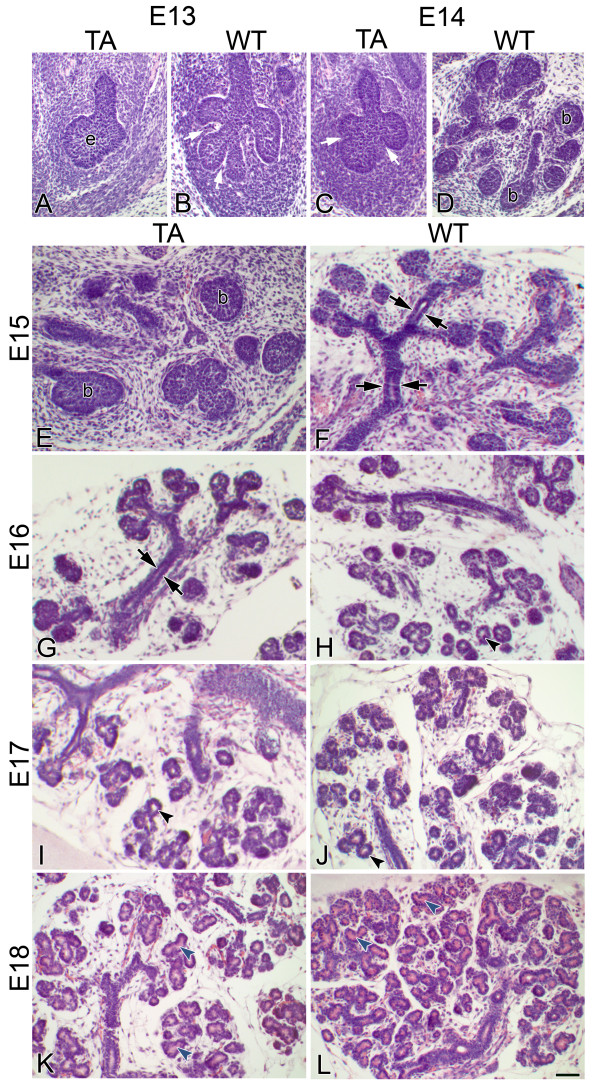
**Embryonic Tabby SMGs are developmentally-delayed**. E13 Tabby (TA) SMG (A) has achieved the *Initial Bud *stage, consisting of a single end bulb. In contrast, E13 wildtype (WT) glands (B) are characterized by cleft formation in the end bud and the formation of a few branches, indicating that it has achieved the *Late Initial Bud *Stage. The *Pseudoglandular *stage, composed of a network of epithelial branches and end buds (b), is seen in E14 WT (D) and E15 Tabby (E) SMGs. The presence of ductal lumina (arrows) indicates that the E15 WT (F) and E16 Tabby (G) SMGs have achieved the *Canalicular *stage. The presence of distinct lumina surrounded by cuboidal epithelia (black arrowhead) in some, but not all, terminal end buds indicates that E16 WT (H) and E17 Tabby (I) SMGs have achieved the *Early Terminal Bud *stage. By E18, differences in branching morphogenesis and glandular maturation are seen between E18 WT (L) and Tabby (K) glands. Note that the E18 Tabby glands (K) are smaller and exhibits fewer branches than E18 WT glands (L); its branching morphogenesis appears similar to that seen in E17 WT (J) glands. However, the observation of distinct proacinar lumina surrounded by a single layer of cuboidal epithelium (blue arrowhead) and continuity between ductal and proacinar lumina in both E18 Tabby (K) and WT (L) glands (i.e. *Late Terminal Bud *stage) indicates the similarity in E18 Tabby and WT maturation. Bar: A-D, 40 μm; E-L, 30 μm.

**Figure 2:**
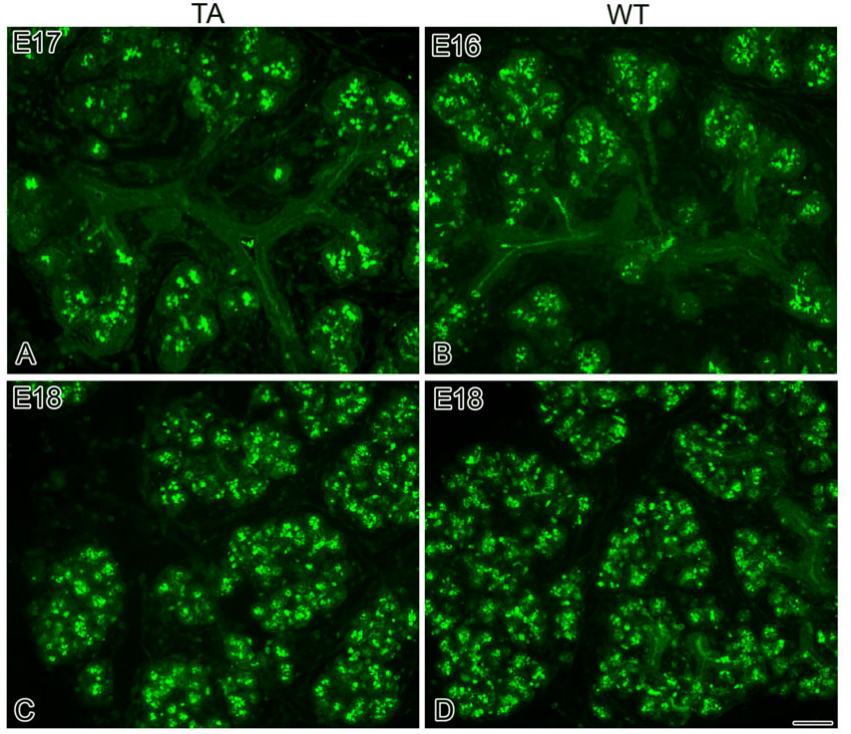
**Mucin protein expression in Tabby and WT glands**. Mucin protein is immunolocalized in some, but not all, terminal end buds in E17 Tabby (A) and E16 WT (B) SMGs (*Early Terminal Bud *stage). By E18, although E18 Tabby SMGs (C) display a notable reduction in total proacini displaying mucin protein compared to E18 WT glands (D), the observation of mucin protein in all proacinar lumen in both E18 Tabby and WT glands indicates similar glandular differentiation and that both glands have achieved the *Late Terminal Bud *stage. Bar, 20 μm.

From the outset, embryonic Tabby SMGs are smaller, exhibit fewer branches and are developmentally delayed compared to WT glands (Fig. 1). At E13, the Tabby gland is characterized by a solid epithelial stalk ending in a bulb and achieving the *Initial Bud *stage (Fig. 1A), whereas the WT gland exhibits deep clefts and the formation of 3–6 epithelial buds (branches) (i.e. *Late Initial Bud *stage) (Fig. 1B). Moreover, although the *Pseudoglandular *stage, composed of a network of epithelial branches and terminal buds, is achieved in E14 WT SMGs (Fig. 1D), the Tabby gland has only progressed to *Late Initial Bud *stage on E14 (Fig. 1C) and does not achieve the *Pseudoglandular *stage until E15 (Fig. 1E). This developmental delay of approximately 1 day persists in Tabby glands, with the *Canalicular *stage being seen in E15 WT and E16 Tabby glands (compare Fig. 1F to 1G) and the Early *Terminal Bud *stage being seen in E16 WT and E17 Tabby glands (compare Fig. 1H to 1I, 2A to 2B). The initial detection of immunolocalized mucin protein in Tabby E17 glands, as well as the similarity of its distribution pattern to E16 WT glands, indicates that Tabby proacinar maturation is also delayed, but not precluded (compare Fig. 2A to 2B). At E18, the Tabby glands remain smaller and exhibit fewer presumptive acini than E18 WT glands (compare Fig. 1K to 1L), a hypoplastic phenotype. Nevertheless, both E18 WT and Tabby SMGs display distinct proacinar lumina bounded by a single layer of cuboidal epithelium and filled with mucin protein, as well as continuity between ductal and proacinar lumina (compare Fig. 1K to 1L, and 2C to 2D). This similarity in E18 Tabby and WT phenotypes indicates that terminal differentiation (presumptive functional maturation) is less affected by the *Eda*^*Ta *^mutation.

### Gene expression

A key principle of signaling and transcriptional regulation of development is that a wide array of ontogenic effects can emerge from a relatively small number of individual molecular components [27]. This nonlinear process is dependent on the functional integration of information transmitted by diverse pathways. Many such pathways in SMG ontogeny have been identified (see reviews, [28-31]). The putative functional relationships within and between pathways have been modeled for mouse SMGs as the estimative equivalent of a "café napkin" sketch, namely a diagrammatic genetic network [28,32].

Mapping genes to their function is called the "genotype-to-phenotype problem," where phenotype is whatever is changed in the organism when a gene's function is altered [33]. Because developmental regulation is both robust and degenerate, it is limiting to simply investigate the average effects of single network genes across samples, large or small [34]. Rather, we are compelled to map genotype to phenotype within the context of the underlying complexity of the networks that regulate cellular functions [33].

**Figure 3:**
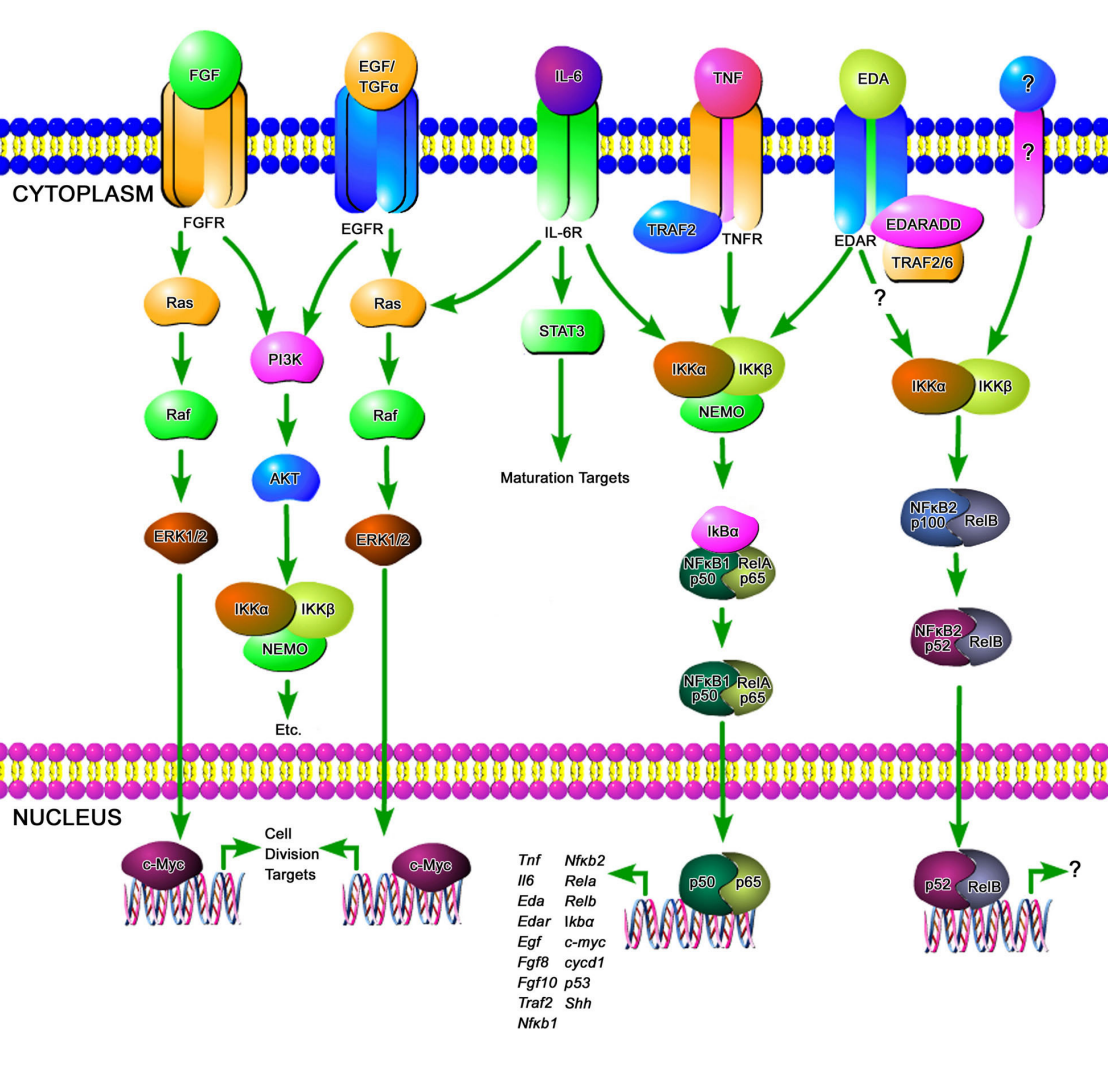
**Subcircuit Map: A relational model that postulates how signaling events likely propagate during SMG development; a conceptionally simple subcircuit for subsequent kinetic modeling (see Additional file **1**and text) that is experimentally constrained and computationally accessible**. This subcircuit is composed of 5 signaling pathways that are both critical to SMG ontogeny and share post-activation downstream targets.

Here we investigated a subcircuit (Fig. 3) that includes 31 probative genes (Tables 1, 2 and 3) in 5 signaling pathways that are both critical to SMG ontogeny and share post-activation downstream targets: Eda, Tnf, Il6, Egf, Fgf [14,28-31,35-42]. This approach has proven very useful, not least because it is experimentally constrained and computationally accessible [43]. Below we present novel emergent properties that would not be otherwise evident.

**Table 1:** Relative Gene Expression (R)*: Wildtype (WT)

**Gene**	**E16**	**E17**	**E18**	**E19**	**NB**
*Eda*	0.32	0.27	0.49	0.74	0.67
*Edar*	0.18	0.10	0.23	0.45	0.67
*Edaradd*	1.00	0.54	0.58	0.52	0.54
*Tnf*	0.09	0.07	0.06	4.98	1.00
*Traf2*	1.00	1.00	1.00	1.34	1.00
*Traf6*	0.74	1.00	1.00	1.45	1.00
*Nfkb1*	0.25	0.17	0.34	1.00	1.00
*Nfkb2*	1.00	1.00	1.00	1.00	2.28
*Rela*	2.28	1.99	2.34	3.64	1.00
*Relb*	0.54	0.47	0.24	1.00	1.00
*Ikkalpha*	1.00	1.00	1.00	1.00	1.00
*Ikkbetta*	1.00	1.00	1.00	1.00	1.00
*Ikbalpha*	1.00	1.44	1.00	2.81	3.28
*Il6*	0.46	0.15	0.26	0.24	1.00
*Shh*	0.32	1.00	1.00	1.00	1.00
*Fgf8*	0.25	1.00	1.00	1.00	1.00
*Fgf10*	29.01	23.84	25.29	0.24	1.00
*Fgfr2*	1.00	1.00	1.00	1.00	1.00
*Egf*	1.00	0.10	0.08	0.02	0.01
*Tgfalpha*	2.60	0.34	0.37	0.18	0.22
*Egfr*	1.47	0.61	0.66	0.40	0.43
*Erk1*	1.00	1.00	1.00	2.42	1.00
*Erk2*	0.65	1.00	1.00	1.00	0.52
*c-myc*	0.51	0.57	0.47	1.00	0.61
*Cyclind1*	1.00	0.67	1.00	0.51	0.33
*Cdk1*	1.00	0.57	1.00	0.42	0.24
*Pi3K*	0.82	1.00	0.86	1.00	1.00
*Akt*	1.00	1.00	1.00	1.00	1.00
*P53*	0.80	1.00	0.49	0.51	0.28
*Casp3*	1.00	1.00	1.00	1.00	0.53
*Stat3*	1.00	1.00	1.00	1.88	1.00

* R = Σr/n, where gene expression (R) is "normalized" to WT-E15 (r = WT-E16/WT-E15, r = WT-E17/WT-E15, etc.), and n ≥ 9. Thus, entries in each cell are mean values (R); values greater or lesser than 1 are significant at P < 0.01 or less.

**Table 2:** Relative Gene Expression (R)*: Tabby (Ta)

**Gene**	**E16**	**E17**	**E18**	**E19**	**NB**
*Eda*	0.06	0.04	0.02	0.07	0.02
*Edar*	0.38	0.03	0.02	0.36	0.14
*Edaradd*	0.76	0.61	0.36	0.33	0.31
*Tnf*	1.00	0.08	0.02	3.90	1.00
*Traf2*	1.00	1.00	1.00	1.00	1.00
*Traf6*	1.00	1.24	1.00	1.00	1.69
*Nfkb1*	1.00	0.13	0.21	1.00	1.13
*Nfkb2*	0.61	1.00	1.00	1.00	1.72
*Rela*	1.00	1.00	2.06	2.03	2.79
*Relb*	0.26	0.29	0.16	0.48	1.00
*Ikkalpha*	1.00	1.00	1.00	2.04	1.00
*Ikkbetta*	1.00	1.00	1.00	3.89	1.00
*Ikbalpha*	1.00	1.00	1.45	1.00	3.13
*Il6*	1.00	0.33	0.11	0.27	0.17
*Shh*	0.19	0.42	0.23	0.24	0.48
*Fgf8*	0.19	0.28	0.40	0.27	0.22
*Fgf10*	7.40	19.54	26.51	0.25	0.17
*Fgfr2*	1.00	0.83	0.83	1.00	1.00
*Egf*	1.00	1.00	1.00	1.00	1.00
*Tgfalpha*	1.00	3.59	4.74	4.20	6.07
*Egfr*	0.77	1.52	1.73	1.48	2.31
*Erk1*	1.00	1.00	2.14	1.00	2.24
*Erk2*	1.00	1.00	1.00	0.64	1.00
*c-myc*	1.00	0.59	0.57	0.37	1.00
*Cyclind1*	1.00	0.79	0.65	0.42	0.51
*Cdk1*	1.00	1.00	0.46	0.47	0.23
*Pi3K*	1.00	1.00	0.76	0.68	0.83
*Akt*	1.00	1.00	1.00	1.00	1.00
*P53*	0.56	0.64	0.43	0.36	0.48
*Casp3*	1.00	0.52	0.55	1.00	0.55
*Stat3*	1.00	1.00	1.40	1.00	2.01

* R = Σr/n, where gene expression (R) is "normalized" to WT-E15 (r = Ta-E16/WT-E15, r = Ta-E17/WT-E15, etc.), and n ≥ 9. Thus, entries in each cell are mean values (R); values greater or lesser than 1 are significant at P < 0.01 or less.

**Table 3:** Relative Gene Expression: Ta/WT

**Gene**	**E16**	**E17**	**E18**	**E19**	**NB**
*Eda*	0.19	0.15	0.04	0.09	0.03
*Edar*	2.11	0.30	0.09	0.80	0.21
*Edaradd*	0.76	1.13	0.62	0.63	0.57
*Tnf*	11.11	1.14	0.33	0.78	1.00
*Traf2*	1.00	1.00	1.00	0.75	1.00
*Traf6*	1.35	1.24	1.00	0.69	1.69
*Nfkb1*	4.00	0.76	0.62	1.00	1.13
*Nfkb2*	0.61	1.00	1.00	1.00	0.75
*Rela*	0.44	0.50	0.88	0.56	2.79
*Relb*	0.48	0.62	0.67	0.48	1.00
*Ikkalpha*	1.00	1.00	1.00	2.04	1.00
*Ikkbetta*	1.00	1.00	1.00	3.89	1.00
*Ikbalpha*	1.00	0.69	1.45	0.36	0.95
*Il6*	2.17	2.20	0.42	1.13	0.17
*Shh*	0.59	0.42	0.23	0.24	0.48
*Fgf8*	0.76	0.28	0.40	0.27	0.22
*Fgf10*	0.26	0.82	1.05	1.04	0.17
*Fgfr2*	1.00	0.83	0.83	1.00	1.00
*Egf*	1.00	10.00	12.50	50.00	100.00
*Tgfalpha*	0.38	10.56	12.81	23.33	27.59
*Egfr*	0.52	2.49	2.62	3.70	5.37
*Erk1*	1.00	1.00	2.14	0.41	2.24
*Erk2*	1.54	1.00	1.00	0.64	1.92
*c-myc*	1.96	1.04	1.21	0.37	1.64
*Cyclind1*	1.00	1.18	0.65	0.82	1.55
*Cdk1*	1.00	1.75	0.46	1.12	0.96
*Pi3K*	1.22	1.00	0.88	0.68	0.83
*Akt*	1.00	1.00	1.00	1.00	1.00
*P53*	0.70	0.64	0.88	0.71	1.71
*Casp3*	1.00	0.52	0.55	1.00	1.04
*Stat3*	1.00	1.00	1.40	0.53	2.01

### WT gene expression

We determined the sequential time-dependent changes in the expression of 31 genes from the earliest time of immunodetectable Eda and Edar to newborn by quantitative RT-PCR. The presentation of wildtype (WT) gene expression data for embryonic day 16 (E16) to newborn (NB) is found in Table 1. The relative expression ratio (*R*) is the mean increase or decrease in gene expression in WT glands compared to a single standard, WT E15 glands, the earliest time of Eda and Edar protein expression. These data are particularly heuristic in our attempt to more fully expand upon what is already known about the control of embryonic SMG branching from prior genotype to phenotype analyses [14,28,30-32,37,38,41,42,44].

Normal *Terminal Bud *Stage (E16-NB) maturation is critical to subsequent acini and ductal terminal differentiation, postnatally. The steady state gene expression of *Fgf8 *and *Fgfr2*, and the more dramatic 20+ fold upregulation of *Fgf10*, suggests that the Fgf pathways are important to *Terminal Bud *Stage maturation in the same way they are prior to this stage. This also may be said of the functionally related *Shh *gene expression. Progression from the *Pseudoglandular *Stage to the *Canalicular *Stage, and continued epithelial branching, also includes Eda/Edar, Tnf and Il6 signaling through the canonical NFκB pathway. The importance of these pathways for *Terminal Bud *Stage maturation appears considerably diminished: *Eda*, *Edar*, *Edaradd*, *Tnf*, *Il6*, and *Nfkb1 *gene expression are significantly (P < 0.01) downregulated. Finally, the Egf/Tgfα/Egfr pathway regulates the rate of branching and histodifferentiation in preparation for progression from the *Canalicular *Stage to the *Terminal Bud *Stage. The steady state of *Egf *expression and the significant upregulation of *Tgfα *and *Egfr *suggest the importance of this signaling pathways through *Early Terminal Bud *Stage (E16); however, beyond this point there is a highly significant (P < 0.01) decline in *Egf*, *Tgfα*, and *Egfr *expression, suggesting a greatly diminished role in SMG maturation.

### Tabby gene expression

To determine if the sequential expression of these important 31 genes differs in Tabby SMGs from that seen in WT SMGs, we also compared Tabby E16 to NB to the same standard (WT E15). The presentation of Tabby SMG gene expression data for E16 to NB, and its comparison to WT is found in Tables 2 and 3. It is readily apparent that embryonic *Eda*^*Ta *^SMGs have many highly significant (P < 0.01) gene expression differences as compared to WT. Such differences have always to be viewed in the context of progressive SMG pathogenesis (Figs. 1, 2).

**Figure 4:**
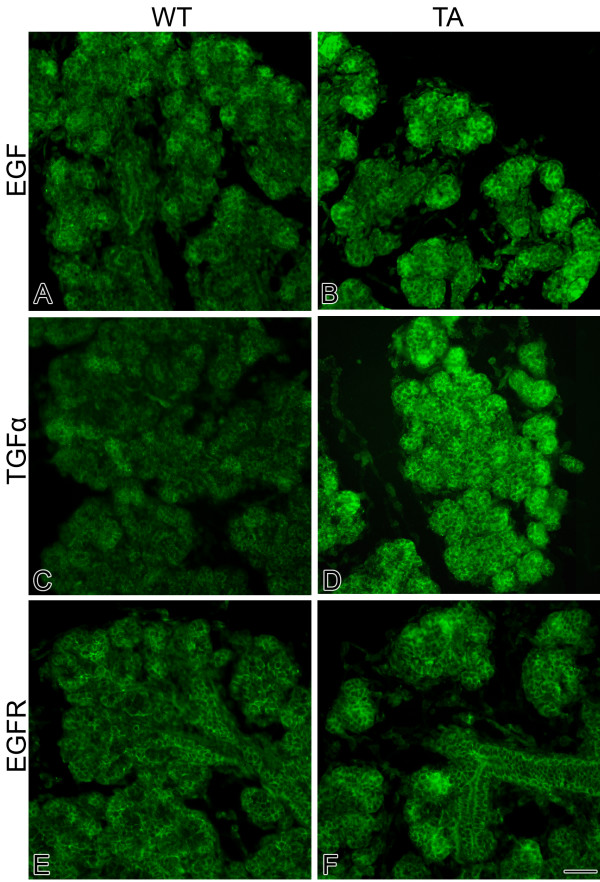
**Tabby SMGs exhibit a marked increase in Egf, Tgfα and Egfr protein expression**. A, B. Immunolocalization of Egf protein in E18 WT (A) and Tabby (B) SMGs. C, D. Immunolocalization of Tgfα protein in E18 WT (C) and Tabby (D) SMGs. E, F. Immunolocalization of Egfr protein in E18 WT (E) and Tabby (F) SMGs. In WT glands, Egf, Tgfα and Egfr proteins are localized to ductal and terminal bud epithelia. In Tabby glands, a substantial increase in immunodetectable Egf, Tgfα and Egfr proteins is seen. Bar, 20 μm.

Though *Eda *gene expression in embryonic *Eda*^*Ta *^SMGs are 3–19% that of WT, in the *Early Terminal Bud *Stage there is a 4-fold increase in *Nfkb1 *gene expression relative to WT (Table 3). This may largely be explained by the concomitant 11-fold increase in *Tnf *expression, Tnf being a powerful inducer of NFκB activation in SMGs [41]. By *Mid *to *Late Terminal Bud *stage, this response is replaced by near normal *Nfkb1 *and *Tnf *expression (Table 3), and dramatically upregulated *Egf*/*Tgfα*/*Egfr *expression: *Egf *by a relative 10–100 fold; *Tgfα *by a relative 10–30 fold, *Egfr *by a relative 2.5–5 fold (Table 3). This unexpected upregulation of *Egf/Tgfα*/*Egfr *message is reflected in the expression of immunodetectable proteins (Fig. 4), with a substantial increase in Egf, Tgfα, and Egfr proteins being seen in Tabby SMGs as compared to WT glands. At the same time, diminished *Eda *expression is associated with significantly (P < 0.01) downregulated *Shh *and *Fgf8 *expression, an outcome previously shown to be correlated with severe SMG pathology [38,44]. Shh and Fgf8 are known to positively and reciprocally regulate one another in SMGs [38]. Still, while *Shh *and *Fgf8 *expression in WT SMGs is highly correlated (P < 0.01) and the variation of one accounts for nearly all of the variation in the other, in *Eda*^*Ta *^SMGs they are not at all correlated (P > 0.90), one accounting for less than 4% of the variation in the other.

In summary, then, the pathology of *Eda*^*Ta *^SMGs, relative to the circumscribed signaling subcircuit (Fig. 3), is associated with downregulated *Shh *and *Fgf8 *gene expression. Perhaps, as a mark of this subcircuit's robustness, the potential severity of the SMG pathology may be mitigated by vastly upregulated *Egf/Tgfα*/*Egfr *gene expression (Table 3) and protein expression (Fig. 4). These results naturally raise questions regarding the underlying mechanism that links the loss-of-function *Eda*^*Ta *^mutation to the positive and negative regulation of the 5 genes just noted (*Shh*, *Fgf8*, *Egf, Tgfα*, *Egfr*), and the overall putative relationship to NFκB-mediated gene regulation.

### Network analysis

The expression of an individual gene in a developing organ is not a soliloquy; rather, it acts in a chorus of quantitative functional relations appropriately termed a genetic circuit, network or connections map. It is the task of systems biology to quantitatively define and analyze the parts (subcircuits) of the whole, the goal being to put it all together in the future [45-49]. To do this, two effective strategies have emerged. One seeks to infer biologic pathways from large data sets and increasingly powerful software tools for managing and searching literature and pathway databases [50]. The second seeks to construct and test increasingly complex mechanistic models of biologic systems [51]. Here we have combined these strategies to determine the way in which a mechanistic pathway diagram (derived from our prior studies, the literature, and pathway databases) can be programmatically transformed to a corresponding system of ordinary differential equations in order to quantitatively test the proposition that the canonical NFκB signaling cascade is sufficient to account for the differential quantitative expression of developmentally regulated genes in the context of *Eda *polymorphism (*Eda *v. *Eda*^*Ta*^).

Pathway and literature databases were mined for processes that interrelate a quantitatively measured panel of genes which are critical to SMG development (Tables 1, 2 and 3; Fig. 3). Mined processes were assembled into a mechanistic kinetic framework and the model was tested for consistency with the WT and Tabby quantitative RT-PCR derived expression data. The resulting best-fit mechanistic system diagram contains 138 states (a state being a molecule or a complex in a physical place) in 5 cellular locations (nucleus, cytoplasm, secretory pathway, plasma membrane and extracellular space), and 217 processes (transport, chemical transformation or binding). The system diagram is too large to be displayed legibly on a journal page, but is provided as a scalable PDF file in Additional file 1, along with access to all the equations and parameters for both WT and Tabby data sets, as well as the fits of the quantitative experimental data (see Additional file 2; Additional file 3; Additional file 4; Additional file 5).

Previous systems analyses have utilized computational methods that allow ready deduction of genetic network connectivity and functional properties solely from gene expression data, temporal or not [52]. Still, recent studies in yeast and E. coli compel the caveat that the ratios of protein levels between mutant and WT may not have a one-to-one correlation with those of the corresponding mRNAs [53,54]. Further, since the mechanistic diagram inevitably includes a large number of proteins as well as expressed mRNAs, we concede at the outset that gene expression data alone are not sufficient to allow highly detailed parameter identification of the downstream protein networks.

Fortunately, the kinetic characteristics of signaling circuits provide general constraints that support quantitative analysis of gene expression data in isolation. First, it is generally agreed that protein transport from the site of synthesis in the ER to the cell surface requires no more than 30 minutes [55]. Second, it is widely acknowledged that signaling pathways require on the order of only two minutes to convey information from the plasma membrane to the nucleus. Since the developmental time courses we analyze include transients on a time scale of 4 to 5 days, we adopted the working hypothesis that signal transduction events (mediated by protein transport in the secretory pathway, receptor binding, and binding to response elements) are fast relative to the dynamics of transcription, mRNA turnover, translation and protein turnover. This simplification allows a full mechanistic test of a complex hypothetical model even in the absence of experimental protein kinetic data on secreted cytokines and growth factors, activated enzymes, and transcription factors (see Methods).

**Figure 5:**
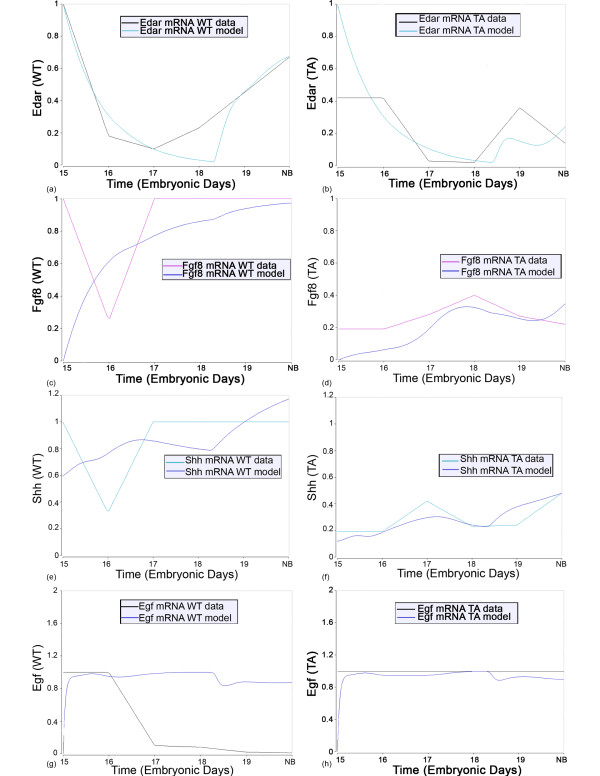
**Time course fits of the WT and Tabby experimental data for a subset of NFκB response genes: *Edar *(a, b); *Fgf8 *(c, d); *Shh *(e, f), *Egf *(g, h)**. The vertical axis is the relative abundance of mRNA, as presented in Tables 1 and 2. The lines labeled "WT data" are the quantitative RT-PCR derived mRNA data in wildtype SMGs; the lines labeled "WT model" are model simulated expected mRNA expression for wildtype SMGs. The lines labeled "TA data" are the quantitatively RT-PCR derived mRNA data in Tabby SMGs; the lines labeled "TA model" are model simulated expected mRNA expression for Tabby SMGs mice.

It is readily apparent that *Eda *is not a *major *determinant of NFκB signaling. This conclusion is inescapable given the large number of genes in the model (Fig. 3; see Additional file 1) whose transcription is reported to be significantly regulated by canonical NFκB. Given our working assumption that all differences between WT and Tabby developmental mRNA profiles are secondary to differential *Eda *expression, it must follow that if Eda is the dominant determinant of Eda/Edar signaling, then the Tabby state of many-fold less Eda must propagate in this kinetic model (see Additional file 1) into much reduced expression of genes whose transcription is significantly regulated by NFκB. To the contrary, only 4 (*Edar*, *Fgf8*, *Shh*, *Egf*) of the 17 genes thought to be NFκB response genes (Fig. 3) have dramatically different time courses in WT and Tabby SMGs (Fig. 5; see Additional file 2). This strongly suggests that *Eda *is not a major determinant of NFκB activation in SMG branching morphogenesis, and presages the existence of an additional Eda-initiated transcriptional control of the 4 genes: *Edar*, *Fgf8*, *Shh*, *Egf*.

**Figure 6:**
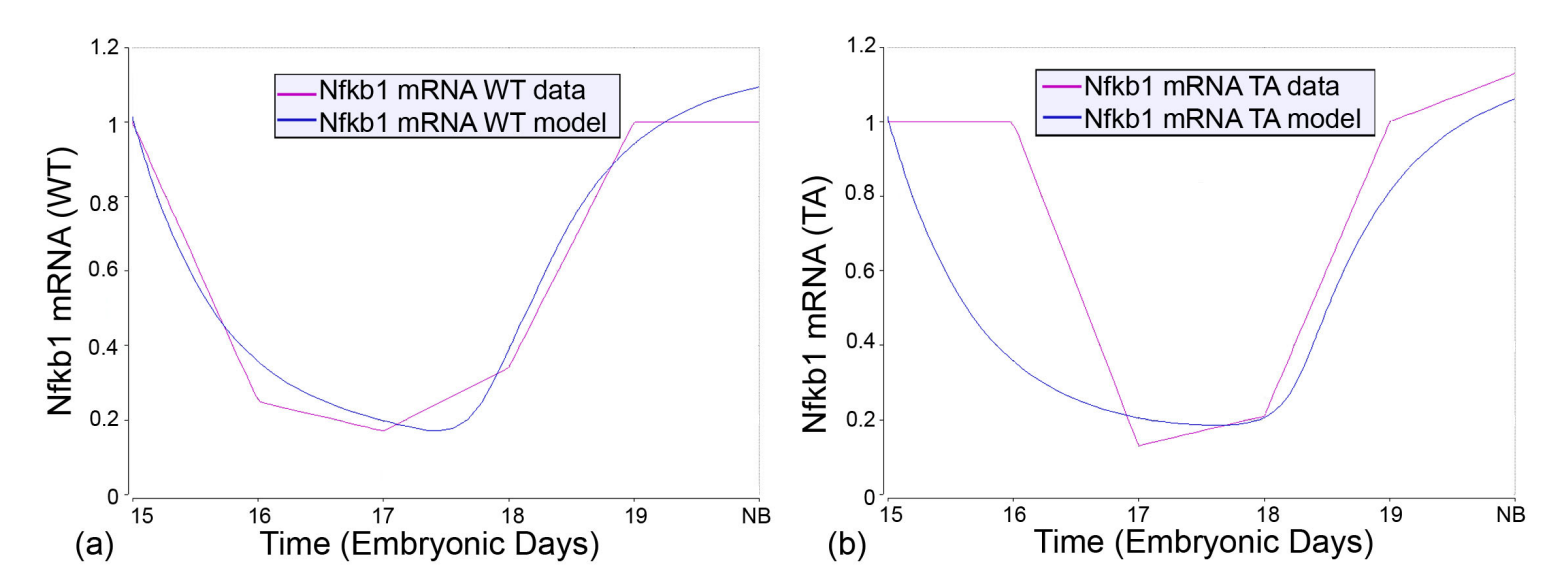
**Time course fits of the WT and Tabby experimental data for *Nfkb1 *gene expression**. The vertical axis is the relative abundance of mRNA, as presented in Tables 1 and 2. The lines labeled "WT data" are the quantitative RT-PCR derived mRNA data in wildtype SMGs; the lines labeled "WT model" are model simulated expected mRNA expression for wildtype SMGs. The lines labeled "TA data" are the quantitative RT-PCR derived mRNA data in Tabby SMGs; the lines labeled "TA model" are model simulated expected mRNA expression for Tabby SMGs mice.

Concomitantly, it is informative to consider *Nfkb1 *gene expression itself. The mechanistic model (see Additional file 1) accounts for the observed significant, but transient, decrease in WT and Tabby SMGs (Tables 1 and 2); *Nfkb1 *expression has essentially the same time course in WT and Tabby SMGs (Fig. 6). Interestingly, the mechanistic model (see Additional file 1; Additional file 3; Additional file 4; Additional file 5) demonstrates that the resulting decrease in protein synthesis is largely buffered by the pre-existing cytoplasmic pool of NFκB1, and thus the downregulation of *Nfkb1 *has little effect on the propagation of Eda, Fgf10, and Tnf induced signals to the nucleus. Further, since prior SMG studies demonstrated that a significant portion of the activation and nuclear translocation of NFκB can be accounted for by the variation in Tnf signaling [32], we tested and corroborated the hypothesis that Tnf, *not *Eda, is the dominant controller of IKK activation in developing WT *and *Tabby SMGs (see Additional file 1; Additional file 3; Additional file 4; Additional file 5).

To address this question further, we employed an *in vitro *loss-of-function strategy to determine if loss of canonical NFκB function abrogates Eda-enhanced SMG branching and maturation. We utilized SN50, a cell permeable inhibitor of NFκB translocation into the nucleus [56], at a concentration (100 μg/ml) previously shown to entirely preclude Tnf- and Eda-induced NFκB nuclear translocation and to significantly inhibit embryonic SMG branching ([32,57], unpublished). To enhance SMG branching, we used 250 ng/ml Eda-A1 in a manner previously reported [14]. Thus, E14 explants were cultured for 7 days (E14 + 7) in the presence of 250 ng/ml Eda-A1, 100 μg/ml SN50, or 250 ng/ml Eda-A1 + 100 μg/ml SN50; controls consisted of E14 explants cultured in control medium alone.

**Figure 7:**
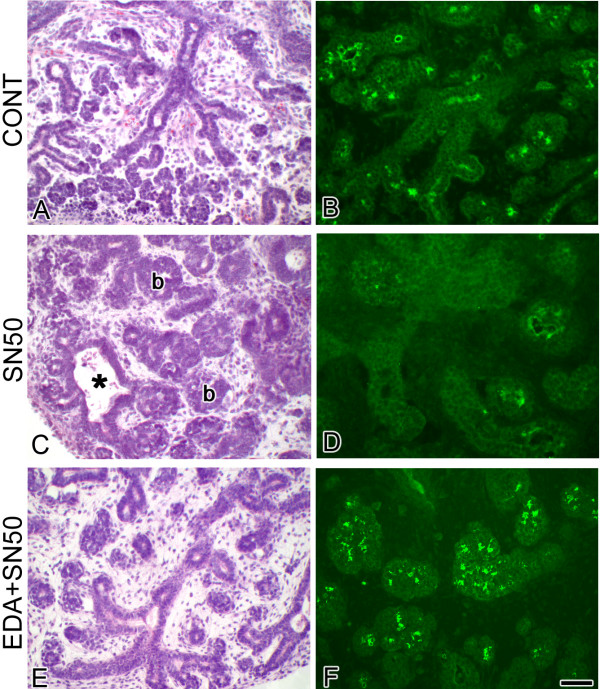
**NFκB function is not essential for Eda signaling**. A, B. E14 + 7 control SMG. C, D. E14 + 7 SMGs cultured in 100 μg/ml SN50. E, F. E14 + 7 SMGs cultured in 250 ng/ml Eda-A1 + 100 μg/ml SN50. A, C, E. Histological analysis. B, D, F. Immunolocalization of mucin protein. SN50 treatment (C, D) induces an abnormal glandular phenotype, characterized by a notable decrease in epithelial ducts and buds (b), dilated ductal lumina (*) and a marked decrease in immunodetectable mucin protein compared to controls (compare C, D to A, B). EDA treatment of SN50-treated explants (E, F) rescues the SN50-induced abnormal phenotype and restores it toward that seen in controls (compare E, F to A, B). The Eda + SN50-treated glands exhibit a marked increase in epithelial ducts and buds, more normally-appearing lumina, and a marked increase in immunodetectable mucin protein compared to glands treated with SN50 alone (compare E, F to C, D). Bar: A, C, E- 40 μm; B, D, F- 25 μm.

Eda supplementation induces a substantial increase (Jaskoll et al., 2003; data not shown) and SN50 induces a marked decrease (compare Fig. 7C, D to 7A, B) in embryonic SMG branching morphogenesis and terminal differentiation, as indicated by mucin protein expression. The presence of exogenous Eda supplementation in SN50-treated explants "rescued" the SN50-induced abnormal phenotype and restored it toward that seen in controls (compare Fig. 7E, F to 7A, B). Note that Eda + SN50-treated glands exhibit a marked increase in epithelial terminal buds, lumina formation, and immunodetectable mucin protein compared to glands treated with SN50 alone (compare Fig. 7E, F to 7C, D). Taken together, our results would indicate that a fully functional NFκB pathway is not the *sine qua non *for Eda signaling, and that Eda signaling likely uses additional, more critical and yet unidentified, pathway(s).

**Figure 8:**
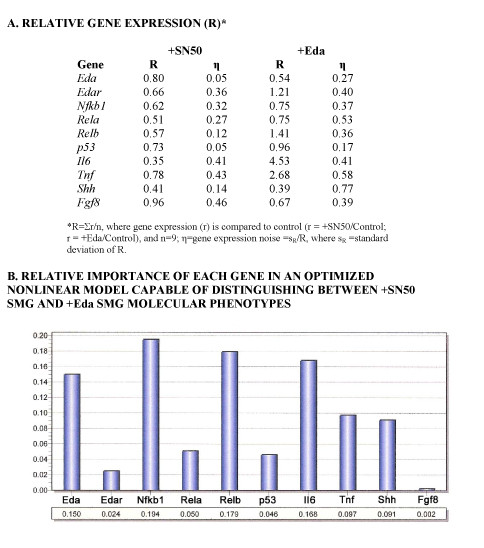
**Comparative *in vitro *gene expression between E14 + 7 SMG explants with NFκB loss-of-function and those with Eda gain-of-function**. A. Quantitative RT-PCR derived relative gene expression in SN50-treated (+SN50) and Eda-treated (+Eda) explants compared to controls. B. Probabilistic Neural Network (PNN) analysis was used to determine the contribution of each gene to the discrimination between SN50-treated and Eda-treated phenotypes. PNN analyses identify the relative importance (0–1, with 0 being of no relative importance and 1 being relatively most important) of specific gene expression changes that distinguish between SN50-treated and Eda-treated phenotypes.

These conclusions were supported by gene expression analysis (Fig. 8). SN50-treated explants show a decline in gene expression of 9 of 10 NFκB1 response genes (Fig. 8A). An optimized (neural network) gene expression model was derived, resulting in a molecular signature that was able to distinguish between SN50-treated and Eda-treated explants with 100% sensitivity and specificity (Fig. 8B). More importantly, when we used the derived model to classify the gene expression data of Eda + SN50-treated explants, we found that *all *Eda + SN50-treated explants (n = 9) were classified as "Eda-treated" and none were classified as "SN50-treated." This is consistent with the histologic "rescue" (Fig. 7E) and again suggests Eda signaling uses pathways in addition to NFκB.

**Figure 9:**
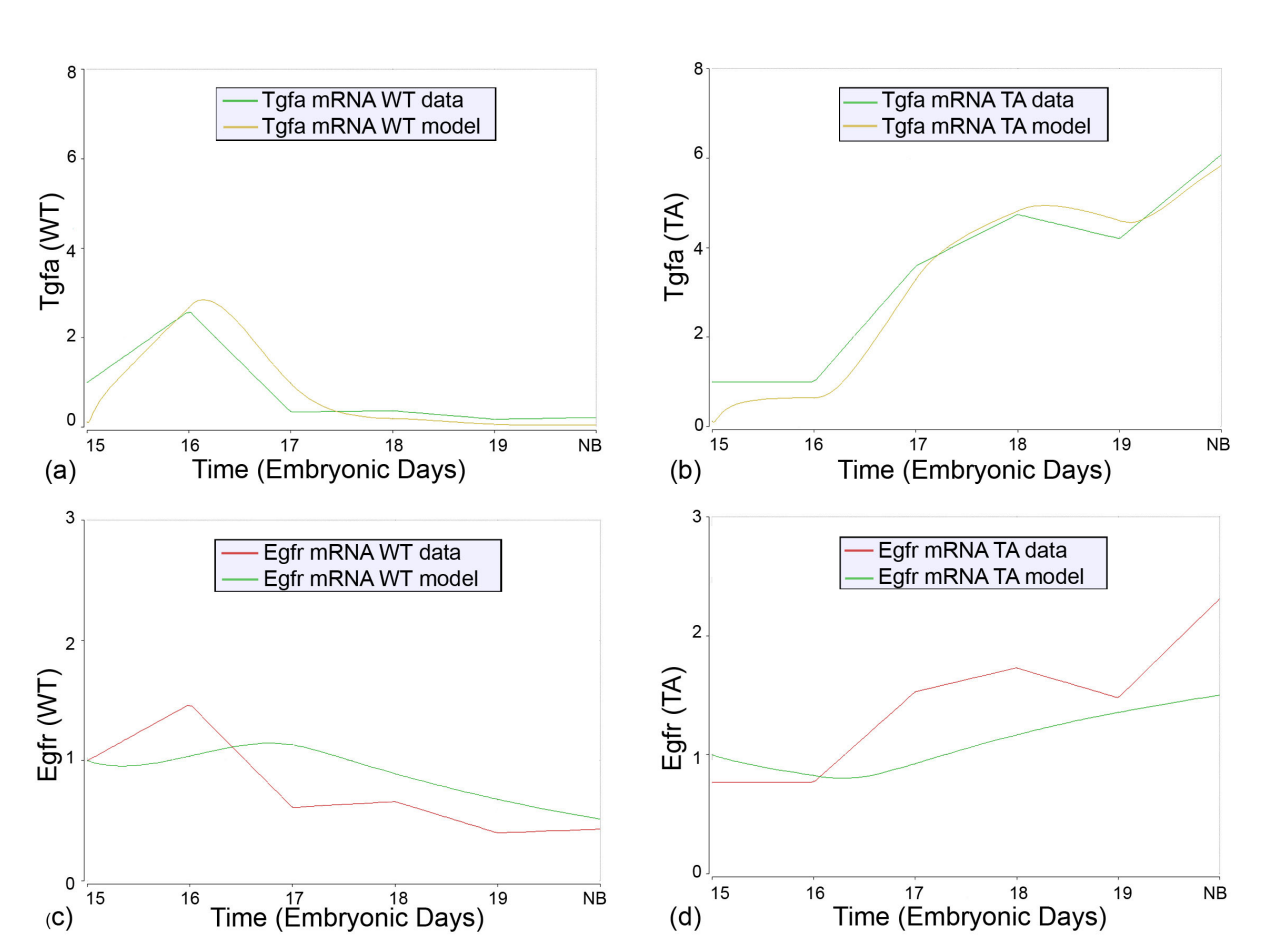
**Time course fits of the WT and Tabby experimental data for *Tgfa *(a, b) and *Egfr *(c, d) gene expression**. The vertical axis is the relative abundance of mRNA, as presented in Tables 1 and 2. The lines labeled "WT data" are the quantitative RT-PCR derived mRNA data in wildtype SMGs; the lines labeled "WT model" are model simulated expected mRNA expression for wildtype SMGs. The lines labeled "TA data" are the quantitative RT-PCR derived mRNA data in Tabby SMGs; the lines labeled "TA model" are model simulated expected mRNA expression for Tabby SMGs mice.

Within our gene expression datasets (Tables 1, 2 and 3), there are four growth factors and two growth factor receptors whose quantitative expression time courses are dramatically different in Tabby SMGs relative to WT: *Egf*, *Tgfα*, *Shh*, *Fgf8*, *Edar*, *Egfr *(Figs. 5, 9). The mechanistic model (see Additional file 1; Additional file 3; Additional file 4; Additional file 5) is internally consistent with some of these but totally incapable of accounting for others. For *Shh *and *Fgf8*, the small differences in nuclear NFκB1 caused by differential Tnf signaling plus the purported feedback of these genes on one another are sufficient to explain the temporal phenotypes of Tabby gene expression reasonably well. For *Egf *and *Edar*, the necessity for a second (non-NFκB) Eda-induced transcriptional regulator is clearly indicated by the discrepancies between model solution and mRNA expression data. For *Tgfα *and *Egfr*, the model solutions are internally consistent but the link to Eda remains unknown. That is, the model can correctly propagate the different temporal phenotypes of WT and Tabby gene expression, but the model cannot explain the causal chain of events that links these genes to Eda. These discrepancies and missing links compelled us to consider an alternative mechanistic explanation and refine the model.

**Figure 10:**
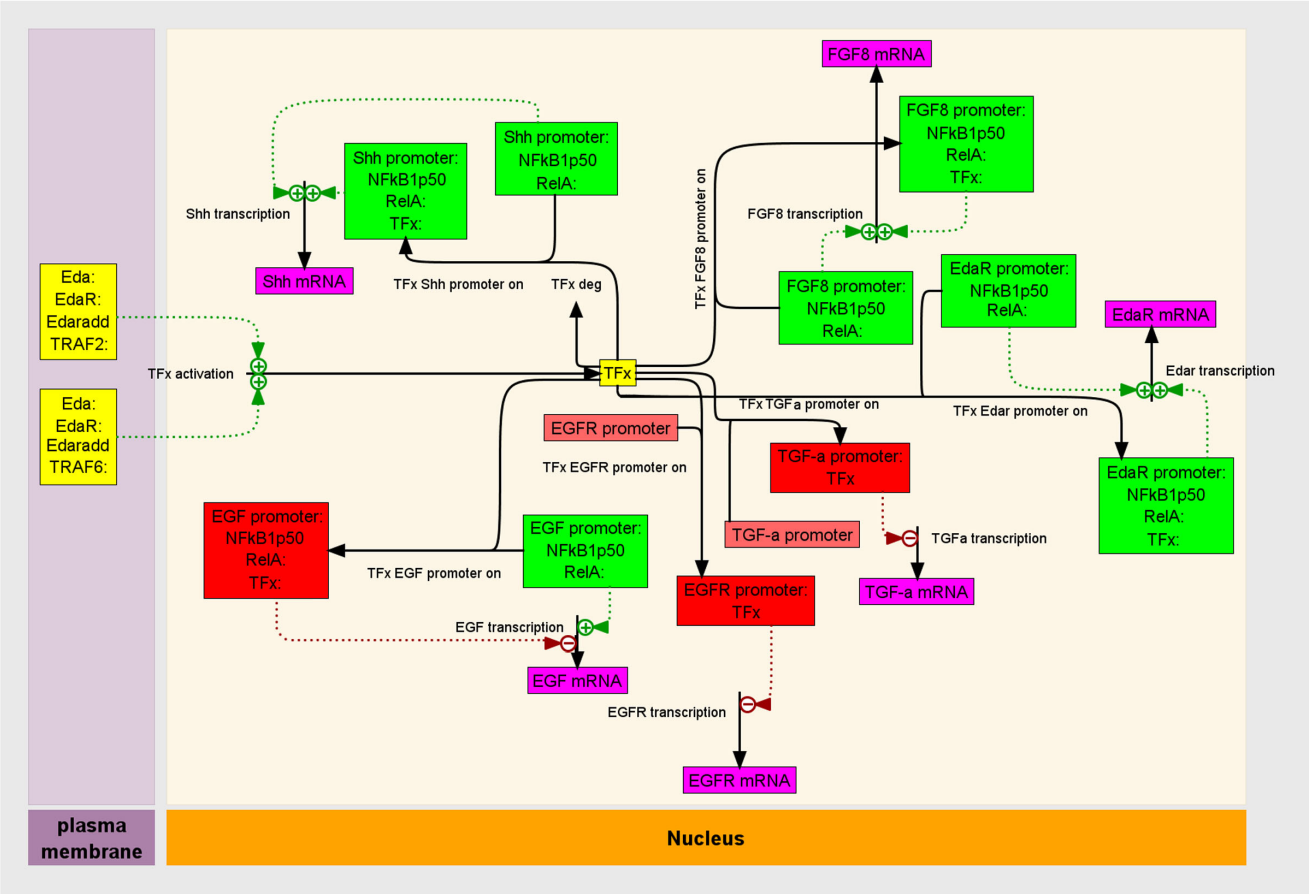
**Alternative kinetic model incorporating unknown transcription factor (TFx) activation by Eda/Edar signaling**. This model is a diagram of the alternative hypothesis that is more consistent with the quantitative mRNA data for Eda/Edar signaling than the hypothesis that NFκB1 serves as the main signaling pathway mediating Eda/Edar signaling. Black arrows represent processes (chemical reactions, transport, or binding). Rectangles represent states. A state is a molecule or complex in a physiologic place. The states are color coded as follows: Yellow is the unknown transcription factor TFx and its upstream activation signaling pathway; purple are the mRNA's; green are the promoters that are upregulated by TFx; red are the promoters that are downregulated by TFx. Places are represented by the background ivory or mauve bands of color and are labeled at the bottom of the diagram. Green and red dashed lines represent, respectively, positive or negative regulation of process by states. Processes with only starts or ends cross the boundary of the modeled system. This diagram was produced by ProcessDB software .

To wit, we hypothesized and tested the alternative model that a second pathway is activated by Eda/Edar signaling, and that its activated transcription factor ("TFx") regulates the six differently expressed genes in unique ways (Fig. 10). With this addition, all six quantitative gene expression time courses are now internally consistent with and accounted for by the mechanistic model. Further, it is possible to deduce whether the added transcriptional regulation is positive or negative. If gene expression is decreased in Tabby SMGs, TFx must be a positive regulator of that gene. Conversely, if gene expression is increased in Tabby SMGs, then TFx must be a negative regulator of transcription. Examination of the quantitative gene expression data (Tables 1 and 2) reveals that there are three of each: *Edar*, *Shh *and *Fgf8 *are positively regulated by the Eda-activated TFx; *Egf, Tgfα*, and *Egfr *are negatively regulated (Fig. 10). The identity of TFx is presently uncertain. However, further *in silico*, *in vivo *and *in vitro *investigations suggest a promising candidate.

### Searching for TFx

**Figure 11:**
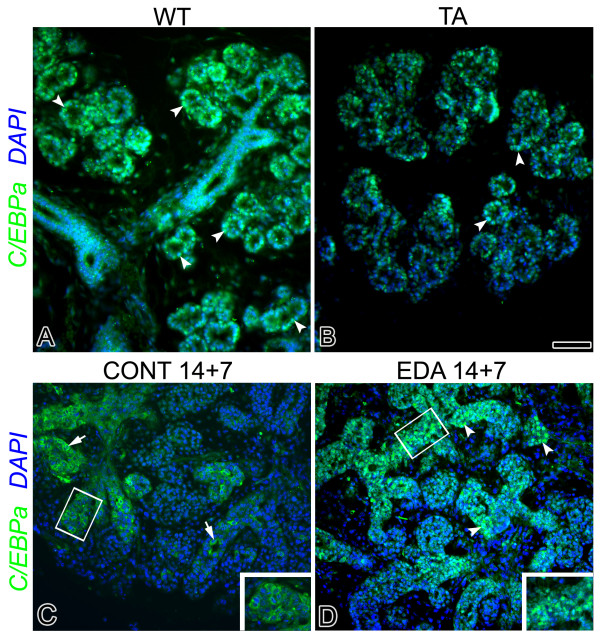
**C/EBPα protein immunolocalization in WT and Tabby glands *in vivo *and in cultured SMGs**. A, B. *In vivo *distribution of C/EBPα protein in E17 WT (A) and Tabby (B) SMGs. C/EBPα protein is primarily localized in nuclei (arrowheads) of epithelial cells surrounding ductal and terminal bud lumina in E17 SMGs. There is a notable decrease in nuclear-localized C/EBPα protein, as well as a marked increase in DAPI-stained nuclei, in Tabby glands (B) compared to WT glands (A). C, D. C/EBPα protein immunolocalization in cultured control (C) and Eda-A1-treated (D) explants. Eda treatment (D) induces a marked increase in immunodectable and nuclear-localized (arrowheads) C/EBPα protein compared to controls (compare D to C). Note that in controls glands (C), C/EBPα protein is found in the cytoplasm whereas the nuclei are labeled with DAPI alone (arrows). Inserts C, D. Higher magnifications showing cytoplasmic-localized C/EBPα protein in controls (C) and nuclear-localized C/EBPα protein in Eda-treated (D) SMGs. Bar, A-B: 20 μm, C-D: 30 μm; C-D inserts: 10 μm.

Regulatory *trans*-acting transcription factors (TF) activate or repress transcription by physical interaction with genomic *cis*-regulating DNA elements that may be found in promoters or at some distance from the target gene's start site(s) (see review, [58]). Putative interactions between TFs and their target DNA sequences can be identified by web-based tools for searching TF binding sites in DNA sequences. Using AliBaba 2.1, TRANSFAC 12.1, MATCH 11.2, and P-MATCH 1.0 (see Methods), we determined that the most probable identity of TFx is CCAAT/enhancer binding protein alpha (C/EBPα). C/EBPα is part of a family of leucine zipper proteins that control the differentiation of many cell types, as well as regulate cell proliferation [59]. Gene and protein analyses are consistent with our *in silico *result and model prediction (Fig. 10). Quantitative RT-PCR reveals a 60% decline in *Cebpa *message in Tabby glands relative to WT at E16, and a 25% decline at E17. In addition, there is a dramatic reduction in activated, nuclear-localized C/EBPα protein in Tabby SMGs relative to WT SMGs at E17 (compare Fig. 11B to 11A). To further delineate the relationship between Eda/Edar signaling and C/EBPα, we compared the spatial distribution of C/EBPα protein in Eda-treated and control E14 + 7 explants (compare Fig. 11D to 11C). Enhanced Eda/Edar signaling *in vitro *induces a notable increase in activated, nuclear-localized C/EBPα protein. Taken together, our *in vivo *and *in vitro *data suggest that C/EBPα is an Eda responsive gene, thus a promising TFx candidate.

The ability of C/EBPα to regulate differentiation and proliferation in a context-specific manner often depends on the presence of specific collaborating TFs [59,60]. Composite *cis*-regulating elements are combinations of two or more TF binding sites with synergistic regulatory action [61]. Our model predicts that 4 key genes (*Shh*, *Fgf8*, *Edar*, *Egf*) would contain NFκB/C/EBPα composite elements (Fig. 10). Scanning all known human 5'-flanking sequences, Shelest et al. [61] found that the most abundant composite elements were of the NFκB/C/EBPα type, including one in the *Egf *sequence and another in the sequence of a TNF receptor superfamily member. Applying the model for composite elements of the NFκB/C/EBPα type [61], we determined that such putative composite elements were also present in the murine sequences of *Shh*, *Fgf8*, *Edar*, and *Egf*.

That C/EBPα is the identity of postulated TFx in the model (Fig. 10) is far from certain. Still, it is an informed hypothesis which can be tested further with protein-protein and protein-DNA interaction assays of high sensitivity and specificity, among other strategies.

## Discussion

In 1875, Charles Darwin presented a meticulous description of what we now know as X-linked Hypohydrotic Ectodermal Dysplasia (XLHED): "I may give an analogous case, communicated to me by Mr. W. Wedderburn of a Hindoo [sic] family in Scinde, in which ten men, in the course of four generations, were furnished, in both jaws taken together, with only four small and weak incisor teeth and with eight posterior molars. The men thus affected have very little hair on their body, and become bald early in life. They also suffer much during hot weather from excessive dryness of their skin. It is remarkable that no instance has occurred of a daughter being affected...though they transmit the tendency to their sons; and no case has occurred of a son transmitting it to his sons" [62].

Patients with X-linked HED have since been shown to also have SMG hypoplasia and variably reduced salivary secretion [19-21,63]. The reduced saliva flow results in dryness of the oral mucosa and predisposes XLHED patients to dental caries and *Candida albicans *infections. Tabby (*Eda*^*Ta*^) is a mouse homologue of human XLHED, a model of the human syndrome that displays near identity. Adult *Eda*^*Ta *^SMGs are hypoplastic with decreased granular convoluted ducts and acini, and there is a notable decrease in immunodetectable mucin protein [14,15]. Prior studies of *in vitro *SMG ontogeny suggested that Eda/Edar signaling effects epithelial cell proliferation, lumen formation, and histodifferentiation via the canonical NFκB pathway [14]. Of great interest, although *Eda*^*Ta *^results in a severely diminished functional Eda ligand [3,4], hemizygosity (males) and homozygosity (females) for the mutant allele result in variable (mild to moderate) adult (mouse and human) SMG hypoplasia, not aplasia [14,19-21,63].

The reproductive success of *mus musculus*, *homo sapiens *and other thriving organisms is in no small measure due to their robustness against perturbations, including gene mutation. This robustness may be seen at all levels of biologic organization from gene expression to terminal organ differentiation and function. What might be the basis of "robustian" rescue (partial or full) from perturbations? Several recent lines of experimental evidence suggest that cells, and their genetic regulatory networks, are dynamically critical [64-68]. Such critical dynamical systems, poised between dynamical order and chaos, maximize the correlated behavior of variables in systems of many variables and maximize the diversity of what they can do as they become larger [69]. In the present study, we sought to detect and measure the degree of SMG developmental robustness, and to elucidate its underlying genetic mechanism.

We began by clarifying the *in vivo *ontogeny of *Eda*^*Ta*^associated SMG pathology (Figs. 1, 2). From the *Initial Bud *stage (E13) on, Tabby SMGs are smaller, exhibit fewer branches and are developmentally delayed compared to WT glands. This is consistent with the findings that in immunodetected in the *Late Pseudoglandular/Early Canalicular *stage (~E15) [14]. Of note, though Eda protein is detectable through the *Late Terminal Bud *Stage (E18-19), comparison of E18 Tabby and WT proacinar phenotypes indicates that terminal differentiation (presumptive functional maturation) is less affected by Eda loss-of-function.

Our prior *in vitro *study [14] clearly showed that control glands express very little activated NFκB, but enhanced Eda/Edar signaling induces a very significant increase in activated NFκB. What we did not show was whether or not canonical NFκB activation was necessary or sufficient for *in vivo *Eda/Edar signaling. We had our doubts because Baltimore's group [70] had shown that *p50*^-/- ^mice have normal development, except for some postnatal (6 weeks) defects in immune responses. Further, we investigated the IkBα transgenic mouse created by Schmidt-Ullrich's group [12,71] and the SMGs are normal (unpublished).

Branching morphogenesis is not a simple dichotomous trait, one we mark present or absent. Rather, it is a complex quantitative trait. Defining the interactions that occur among the genes that underlie the process of branching is essential to understanding its variability. When a gene that is critical to this developmental process mutates, the differentiating cells reprogram transcription via a cognate genetic circuit, ultimately altering the expression of the many genes beyond the mutated one. As such, the phenotype of a given genotype cannot be simply predicted by the sum of its component single-locus effects, but must take account of the almost certain epistasis in gene function [72]. The most efficient way of doing this is by systems analysis, correlation modeling for hypothesis generation (e.g. [32,73,74]) and kinetic (mechanistic) modeling for hypothesis testing [46,48,49,75,76].

The molecular pathology of embryonic Tabby (*Eda*^*Ta*^) SMGs is characterized by significantly (P < 0.01) downregulated *Shh *and *Fgf8 *gene expression, on the order of 50–75% less than WT. Typically, this would be expected to be associated with a severely hypoplastic to aplastic gland [38,44], but this is not the case ([14]; Figs. 1, 2). One obvious explanation for this is the many-fold upregulation of *Egf*/*Tgfα*/*Egfr *gene (Table 3) and protein (Fig. 4) expression. This dramatic robustness of the studied subcircuit (Fig. 3; see Additional file 1) is largely due to degeneracy, namely the ready availability of multiple parallel pathways at a key "choice point [34]. Mechanistic modeling reveals that the primary linchpin of this degeneracy is not canonical NFκB as previously supposed [14]; rather, we provide extensive quantitative evidence that the Eda/Edar/NFκB cascade plays a minor role, apparently neither necessary nor sufficient for SMG development. We confirm this with an *in vitro *strategy that demonstrates that loss of NFκB function does not abrogate Eda-enhanced SMG branching and differentiation (Figs. 7, 8). These outcomes add support to the suggestion by Pispa et al [77] of an important NFκB-independent pathway downstream of Eda/Edar signaling *in vivo*.

The most parsimonious alternative explanation is a second Eda-activated transcription factor ("TFx") that regulates, alone or complexed with NFκB, the expression of all five target genes: *Shh*, *Fgf8*, *Egf*, *Tgfα*, *Egfr *(Fig. 10). Our initial *in silico *and *in vivo *investigations suggest that the identity of TFx is C/EBPα, a regulator of cell proliferation and differentiation [59]. There are likely other, as yet unknown, TFs in the Eda/Edar pathway. Unmasking TFx with certainty, and delineating its participation in transcription factor complexes, could prove formidable [78]; delineating the stochastic variation in cognate gene expression more so [79].

The variable phenotypic expression of human hypohydrotic ectodermal dysplasia is considerable ([22,80]; Melnick unpublished). This is particularly so for glandular phenotypes (salivary, sweat, sebaceous, lacrimal, mammary, and mucous). If branching epithelia use variations of a canonical developmental program, the experimental results presented here should be applicable to understanding the variable phenotypic expression of other branching organs affected by *Eda *(*EDA*) mutation.

## Conclusion

Our prior studies of *in vitro *SMG branching morphogenesis suggested that Eda/Edar signaling largely regulates ontogeny through the canonical NFκB pathway [14]. The present *in vivo *quantitative systems analyses indicate that this conclusion must be amended. The need to do so is inescapable because, for most NFκB-regulated genes, the observed time course of gene expression is nearly unchanged in Tabby mice as compared to wildtype mice (see Additional file 2), as is NFκB itself (Fig. 6). Importantly, a subset of genes is dramatically differentially expressed in the Tabby mouse (*Edar*, *Fgf8*, *Shh*, *Egf, Tgfa*, *Egfr*) (Figs. 5, 9), strongly suggesting the existence of an alternative Eda-mediated transcriptional pathway pivotal for SMG branching morphogenesis (Fig. 10). Experimental and *in silico *investigations have identified C/EBPα as a promising candidate (Fig. 11). Finally, it should be noted that upregulation of the Egf/Tgfα/Egfr pathway appears to mitigate the potentially severe abnormal phenotype predicted by the downregulation of Fgf8 and Shh. It has recently been suggested by Harris et al. that the buffering of the phenotypic outcome that is coincident with variant Eda signaling could be a common mechanism that permits viable and diverse phenotypes, including those we would consider normal [81]. Our results support this proposition.

## Methods

Wildtype (WT) mice were either B6CBACaF1-A^W-J^/A (A^W-J^) or B10.A/SgSn obtained from Jackson Laboratories (Bar Harbor, ME) and bred as previously described (Jaskoll and Melnick, 1999; Jaskoll et al., 2003); plug day = day 0 of gestation. Tabby breeding pairs [B6CBACA A^W-J/^A-Eda^Ta^/O/J (Ta/0) and B6CBACA A^W-J/^A-Eda^Ta^/Y (Ta/Y)] were obtained from Jackson Laboratories and kept by breeding Ta/0 females to Ta/Y males. All embryos from the cross were either Ta/0 or Ta/Ta females and Ta/Y males and displayed the Tabby phenotype. The A^W-J ^wildtype SMGs were used for all comparisons with Tabby glands. E13-19 Tabby and WT pregnant females and newborn mice were sacrificed, SMGs were dissected in cold phosphate-buffered saline (PBS) and glands were collected for morphological, immunolocalization or quantitative RT-PCR. For histological analysis, SMGs were fixed for 4 hrs in Carnoy's fixative at 4°C or overnight in 10% neutral buffered formalin at room temperature, embedded in low melting point paraplast, serially-sectioned at 8 μm and stained with hematoxylin and eosin as previously described [37]. For each embryonic day from gestation day 13 to 18, 5–15 WT and Tabby SMGs were analyzed.

For *in vitro *culture experiments, B10A/SnSg mice were mated and pregnant females were sacrificed on day 14 of gestation (E14) as previously described [14,44] Embryonic SMGs were dissected under sterile conditions and *in vitro *experiments conducted as outlined below.

All animal studies were conducted with the approval of the appropriate committees regulating animal research. An Animal Review Board and a Vivaria Advisory Committee review all applications to ensure ethical and humane treatment.

### Immunolocalization

E16-18 Tabby and WT (A^W-J^) glands were fixed for 4 hrs in Carnoy's fixative at 4°C or overnight in 10% neutral buffered formalin at room temperature, embedded in low melting point paraplast, serially sectioned at 8 μm and immunolocalization was conducted essentially as previously described [14,38,82] using the following affinity-purified antibodies: polyclonal rabbit anti Muc10 [82,83], polyclonal rabbit anti C/EBPα (sc-61; Santa Cruz Biotechnology), polyclonal rabbit anti Egfr (sc-03; Santa Cruz Biotechnology), polyclonal goat anti Egf (sc-1343; Santa Cruz Biotechnology) and monoclonal mouse anti Tgfα (sc-36; Santa Cruz Biotechnology). In selected experiments, nuclei were stained with 4,6-diamidino-2-phenylindole (DAPI). For mucin protein localization, 3–4 WT and Tabby glands were analyzed for each embryonic day and 3–6 explants per treatment group were analyzed. For C/EBPα protein localization, 5 WT or Tabby glands were analyzed and 3–5 explants per treatment group were analyzed. For Egfr, Egf and Tgfα protein localization, 3–4 WT and Tabby glands were analyzed.

### Quantitative RT-PCR

For analysis of gene expression, quantitative RT-PCR was conducted as previously described [57]. WT and Tabby SMGs were pooled (WT-E15-17: 6–20 SMGs/sample; E18-NB: 2–4 SMGs/sample; Tabby-E16-17:15–20 SMGs/sample; E18-NB-4-6 SMGs/sample). For each embryonic day, in each of WT and Tabby, we performed quantitative RT-PCR on 9 or more independent samples. RNA was extracted and 1 μg RNA was reverse transcribed into first strand cDNA using ReactionReady™ First Strand cDNA Synthesis Kit: C-01 for reverse transcription (Superarray Biosciences, Frederick, MD). The primer sets used were prevalidated to give single amplicons and purchased from Superarray Biosciences: Eda (#PPM40603E); Edar (#PPM32386A); Edaradd (PPM32128E); Fgf8 (#PPM02962C); Fgf10 (PPM0345A); Fgfr2 (PPM03706E); PI3k (PPM03469A); Akt (PPM03377A); ERK1 (PPM03575A); ERK2 (PPM03571A); Stat3 (PPM04643E); Egf (PPM03703C); Tgfa (PPM03051A); Egfr (PPM03714E); Ikbkb (PPM03198A); Nfkb1 (#PPM02930A); Nfkb2 (PPM03204A); Rela (#PPM04224E); Relb (#PPM03202C); Tnf (#PPM03113E); Traf2 (PPM03083E); Traf6 (PPM03082A); Il6 (#PPM03015A); p53 (#PPM02931A); Shh (#PPM04516B); cyclin D1 (PPM02903A); Cdk1 (PPM02907A); Casp3 (PPM02922E); Myc (PPM02924A); Ikka (PPM03197A); Ikbkap (PPM37360A); Cebpa (PPM04674A). Primers were used at concentration of 0.4 microM. The cycling parameters were 95°C, 15 min; 40 cycles of (95°C, 15 sec; 55°C, 30–40 sec and 72°C, 30 sec). Specificity of the reactions was determined by subsequent melting curve analysis. RT-PCRs of RNA (not reverse transcribed) were used as negative controls. GAPDH was used to control for equal cDNA inputs and the levels of PCR product were expressed as a function of GAPDH. The relative fold changes of gene expression between the gene of interest and GAPDH, or between the WT and Tabby, were calculated by the 2^-ΔΔCTCT ^method.

### Kinetic modeling and hypothesis testing

#### Data mining

Pathway and literature databases were mined for processes that interrelate a panel of pivotal genes in SMG development (Fig. 3; Table 1). We extracted relevant interactions and pathways from KEGG , OMIM , GeneCards , three models from the BioModels database , iHOP , and the scientific literature accessed through the PubMedCentral database . These data were manually curated and entered into the ProcessDB software . In some cases pathways were available in SBML  format and these were programmatically imported from the BioModels.net database into the ProcessDB database for re-use. The resulting mechanistic system diagram contains 138 states (a state is a molecule or a complex in a physical place) in 5 cellular locations (cytoplasm, nucleus, secretory pathway, plasma membrane and extracellular fluid), and 217 processes (transport, chemical reaction, or binding). It is too large to be displayed legibly on a journal page, but is provided as a scalable PDF file (see Additional file 1).

#### Kinetic analysis and modeling

Experimental mRNA expression data on the genes listed in Table 1 were collected as described above and provided mRNA data on E15, E16, E17, E18, E19, and newborn (NB) for wild type (AW) embryos and on E16, E17, E18, E19, and NB for Tabby embryos whose glands are uniformly and significantly smaller. For each gene analyzed the data were normalized to the value obtained on E15 in the wild type animals. The defining characteristic of the Tabby mouse is an X-linked mutation that causes a major reduction in Eda mRNA. The underlying goal of the modeling work was to understand how this marked reduction in Eda propagates through the signal transduction and genetic networks to produce the observed temporal phenotypes for the measured panel of developmental genes.

A standard biophysical/bioengineering approach to kinetic analysis of a complex system containing many feedback and feedforward controls is to open the control loops by use of forcing functions [84,85]. We took advantage of the rich data set of measured mRNA time courses by using them as forcing functions for protein synthesis in the large-scale mechanistic kinetic model. This approach accelerates model development by propagating data-determined (and presumably correct) time courses through the network even before a consistent model has been obtained. Because Tabby glands are too small at ED15 to be practical for RT-PCR-based measurements, some method of extrapolation back from ED16 to ED15 was required for the Tabby forcing functions. Two methods were possible: 1) assume the value at ED15 is equal to the value at ED16 or 2) assume the slope determined by values at ED16 and ED17 is the same as the slope between ED15 and ED16. Choice 1 was seen as more conservative and was implemented. Rate law parameters and initial abundances were chosen throughout the protein network to propagate the main features of each mRNA developmental transient as faithfully as possible. Default rate laws, based on mass action kinetics were created automatically by ProcessDB for each process. This automatically produces saturation behavior for processes limited by the abundance of one or more reactants. Enzyme catalyzed processes and processes activated or inhibited by other states in the model were supplied with rate laws based on classical rapid equilibrium enzyme kinetics [86].

Another useful modeling technique is, wherever feasible, to analyze multiple experiments simultaneously using the same mechanistic model. This could be implemented in the ProcessDB software, even though the present experiments are performed in two different mouse strains, by adopting the working hypothesis that WT and Tabby mice are identical except for the genetic defect in the Eda gene of the Tabby mutant. In other words, all differences between WT and Tabby developmental mRNA profiles are assumed to be secondary to the difference in Eda expression. In some cases fitting the WT and Tabby data sets required that states have different initial numerical values at ED15. This was allowed because the cytokine environment of the salivary gland in early embryonic development is undoubtedly different in the two strains. In all cases, however, parameter values and rate laws were taken as identical for the two strains. This is an extremely powerful modeling constraint. It does not mean, of course, that all unmeasured protein time courses are the same in the WT and Tabby models. Each is driven by the measured mRNA abundances so that known differences are propagated through common rate laws with the objective of testing the structure of the network – from cytokine production and secretion to receptor binding to activation of enzymes and transcription factors, to nuclear localization and activation or inhibition of cognate genes.

Importantly, the use of mRNA forcing functions in no way guarantees that the full model will simultaneously fit the mRNA data that serve as forcing functions. This is because the control of nuclear transcription factors is, in general, multifactorial and mechanistically distant from synthesis of the proteins involved. Consequently, it was possible to test the mechanistic model's ability to account for the experimental data by searching for transcription and mRNA degradation parameters able to fit the WT and Tabby data simultaneously. The full mechanistic model file including all differential equations, ancillary algebraic equations, rate laws and parameters is included in Additional files (see Additional file 1; Additional file 2; Additional file 3; Additional file 4; Additional file 5).

All differential equations and ancillary algebraic equations were formulated in ProcessDB and exported to the Berkeley Madonna  solver for numerical integration and parameter optimization using standard methods. Assembled pathway diagrams were treated as hypotheses and tested against the WT and Tabby experimental data.

#### Transcription factor analysis

The mouse sequences of *Fgf8*, *Shh*, *Eda*, *Tgfa*, *Egf*, and *Egfr *were analyzed for the presence of putative TF DNA binding sites that are common to all 6 genes. We began with an unbiased search with AliBaba 2.1 , the most specific tool for predicting TF binding sites in an "anonymous" DNA sequence using the TRANSFEC database of TFs. The outcome was manually curated based upon mined pathway and literature databases (see Kinetic Modeling/Data mining above) and rank ordered. This analysis revealed SP1 and C/EBPα to be the most likely candidates. We then analyzed both candidates with greater stringency using MATCH 11.2 (proprietary; ) and P-MATCH 1.0 .

MATCH 11.2 is a weight matrix-based tool for searching putative TF binding sites in DNA sequences [87]. MATCH 11.2 uses the matrix library collected in TRANSFAC 12.1 (proprietary; ). Multiple sets of optimized matrix cut-off values are built into the tool to provide a variety of search modes of different stringency. The matrix similarity is a score (0–1) that describes the quality of a *match *between matrix and an arbitrary part of the input sequence. Analogously, the core similarity score (0–1) denotes the quality of a *match *between the core sequence of a matrix (the five most conserved positions within a matrix) and a part of the input sequence. A *match *has to contain the core sequence of a matrix, i.e. the core sequence has to match with a score higher than or equal to the core similarity cutoff. In addition, only those *matches *which score higher than or equal to the matrix similarity threshold appear in the output. Cut-offs were chosen to minimize false positive and false negative outcomes. Of the two candidate TFs revealed by AliBaba 2.1, only C/EBPα survived the more rigorous analysis.

This outcome was confirmed using P-MATCH 1.0. P-MATCH combines weight-matrix and pattern matching analytical strategies, thus providing higher accuracy of recognition than either method alone [88]. P-MATCH 1.0 uses the library of mononucleotide weight matrices from TRANSFAC 6.0 (public) along with the site assignments associated with the matrices. Comparisons with MATCH, show that P-MATCH generally provides enhanced recognition accuracy vis-à-vis lower false negative errors (i.e. high sensitivity).

#### EDA-A1 supplementation *in vitro*

To determine the optimal concentration of exogenous soluble human recombinant EDA-A1 that induces a significant increase in SMG branching and morphogenesis, we conducted a dose response study. This dose response study was conducted because the EDA-A1 recombinant set (EDA-A1 + enhancer molecule) (Alexis Biochemicals, Axxora, LLC, San Diego, CA) in the present set of experiments was different from the EDA-A1 peptide employed in our previous study [14]. A stock solution of 1 Φg/ml EDA-A1 (10 Φl EDA-A1 + 5 Φl Enhancer + 985 Φl BGJb containing 1% BSA) was made following the manufacturer's protocol and then diluted to 100, 250 and 500 ng/ml. Paired E14 SMG primordia were cultured for 2 days (E14 + 2) and 5–7 days in the presence or absence of 100, 250 and 500 ng/ml EDA-A1. For E14 + 2 explants, Spooner branch ratios (epithelial bud number on day 2/bud number on day 0) were calculated for each explant, comparisons made between right and left glands (treated and control) from each embryo and mean Spooner ratios determined as previously described [14,38,44]. The data were arcsin transformed and compared by paired *t*-test for all embryos studied [89]. Since branching morphogenesis is too complex to count in E14 explants cultured 5–7 days, the morphology of these explants were analyzed by routine hematoxylin and eosin histology; 4–6 explants per dose were analyzed for each day of culture. We determined the optimal dose to be 250 ng/ml EDA-A1 and this concentration was used in all subsequent experiments.

#### SN50 inhibition of canonical NF6B nuclear translocation

The cell permeable peptide SN50 (Biomol Research, Plymouth Meeting, PA) has been shown to inhibit translocation of the canonical NFκB1/RelA pathway into the nucleus [32,56,90]. To determine the effect of SN50 on E14 SMG morphogenesis, paired E14 SMG primordia were cultured for 2 days or 5–7 days in the presence or absence of 100 *μ*g/ml SN50. This concentration was previously shown in our laboratory to be the optimal inhibitor of NFκB1/RelA translocation and interrupts SMG morphogenesis [32,57]. The morphology of these explants was analyzed by routine hematoxylin and eosin histology; 4–6 explants per treatment group were analyzed.

#### *In vitro *rescue experiments

To determine if exogenous EDA-A1 rescues SN50-induced abnormal phenotypes, E14 SMGs were cultured for 7 days in the presence of 250 ng/ml EDA-A1, 100 *μ*g/ml SN50 or 250 ng/ml EDA-A1 + 100 *μ*g/ml SN50; control SMGs consisted of explants cultured in control medium. E14 + 7 explants were collected for histological analysis or quantitative RT-PCR as described above. The morphology of these explants was analyzed by routine hematoxylin and eosin histology; 20–25 explants per treatment group were analyzed. For RT-PCR, 9 independent samples per treatment of pooled glands (20–25 SMGs/sample/group) were analyzed.

#### Probabilistic neural network (PNN) analysis

We used PNN analyses to determine the contribution of each individual gene to the discrimination between experimental groups with 100% sensitivity and specificity. As such, PNN analyses identify the relative importance (0–1, with 0 being of no relative importance and 1 being relatively most important) of specific gene expression changes that discriminate between phenotypes. It is the contextual change in expression, not the direction of change that is important in defining the molecular phenotype. The foundational algorithm we used is based upon the work of Specht and colleagues [91-93]. The proprietary software designed by Ward Systems Group (Frederick, MD) formulates Specht's procedure around a genetic algorithm [94]. A genetic algorithm is a computational method modeled on biologic evolutionary processes that can be used to find the optimum solution to a problem that may have many solutions [95]. These algorithms have been found to be very powerful in solving optimization problems that appear to be difficult or unsolvable by traditional methods. They use a minimum of information about the problem and they only require a quantitative estimation of the quality of a possible solution. This makes genetic algorithms easy to use and applicable to most optimization problems.

## Authors' contributions

TJ and MM conceived and designed the study. MM was involved in and coordinated all experiments, as well as the modeling, and drafted the manuscript. TJ participated in RT-PCR analyses, analyzed the histological and localization data, conducted the *in vitro *experiments, and helped draft the manuscript. RP and SL participated in kinetic modeling and helped draft the manuscript. All authors read and approved the final manuscript.

## Supplementary Material

Additional file 1 **Mechanistic gene network model**. Diagram of the full mechanistic gene network model. Black arrows represent processes (chemical reactions, transport, or binding). Rectangles represent states. A state is a molecule or complex in a physiologic place. Places are represented by the background ivory or mauve bands of color and are labeled at the bottom of the diagram. Green and red dashed arrows represent, respectively, positive or negative regulation of processes by states. Processes with only starts or ends cross the boundary of the modeled system. This diagram and corresponding computational model were produced by ProcessDB software . Click here for file

Additional file 2 **Additional time course fits of the WT and Tabby experimental data by the full mechanistic model**. Time course fits of the WT and Tabby experimental data by the full mechanistic model that are not included in Figures 4, 5, 8 of the main text. Note, *Eda *is not included among the genes because differences in *Eda *expression in WT and Tabby mice are not attributable to transcriptional control. The vertical axis is the relative abundance of mRNA, as presented in Tables 1 and 2. The lines labeled "WT data" are the quantitative RT-PCR derived mRNA data in wildtype SMGs; the lines labeled "WT model" are model simulated expected mRNA expression for wildtype SMGs. The lines labeled "TA data" are the quantitative RT-PCR derived mRNA data in Tabby SMGs; the lines labeled "TA model" are model simulated expected mRNA expression for Tabby SMGs mice. Click here for file

Additional file 3 **Corresponding equations of the computational model**. Text file displaying the corresponding equations of the computational model shown in Additional file 1. Click here for file

Additional file 4 **A Berkeley Madonna model file modified slightly from the model file generated automatically by ProcessDB**. A Berkeley Madonna  model file (MODE3680_20080326_final.mmd) containing all the equations and parameters for both WT and Tabby experiments, as well as the fits of the experimental data. This file can be run and examined using the free trial version of Berkeley Madonna. Click here for file

Additional file 5 **Berkeley Madonna variable names corresponding to those of the model diagram**. Table showing Berkeley Madonna variable names corresponding to those of the model diagram shown in Additional file 1. Click here for file
