# Supplementary figures and images for: Salivary gland branching morphogenesis: a quantitative systems analysis of the Eda/Edar/NFκB paradigm
Source: BMC Dev Biol. 2009 Jun 6;9:32. doi: 10.1186/1471-213X-9-32 (PMC2700095; doi:10.1186/1471-213X-9-32)

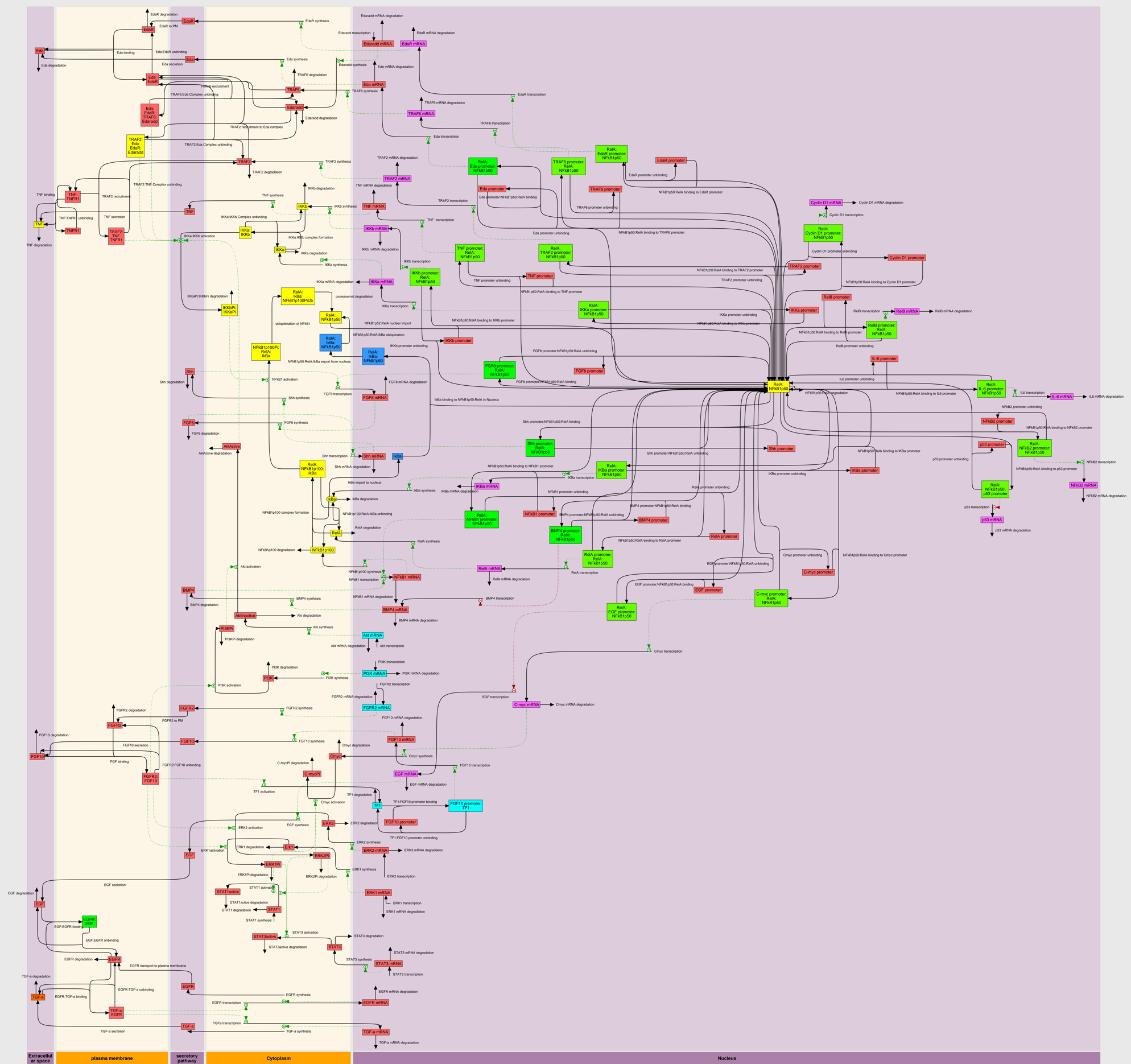

Supplement: Additional file 1 — Mechanistic gene network model. Diagram of the full mechanistic gene network model. Black arrows represent processes (chemical reactions, transport, or binding). Rectangles represent states. A state is a molecule or complex in a physiologic place. Places are represented by the background ivory or mauve bands of color and are labeled at the bottom of the diagram. Green and red dashed arrows represent, respectively, positive or negative regulation of processes by states. Processes with only starts or ends cross the boundary of the modeled system. This diagram and corresponding computational model were produced by ProcessDB software . [file 1471-213X-9-32-S1.pdf]

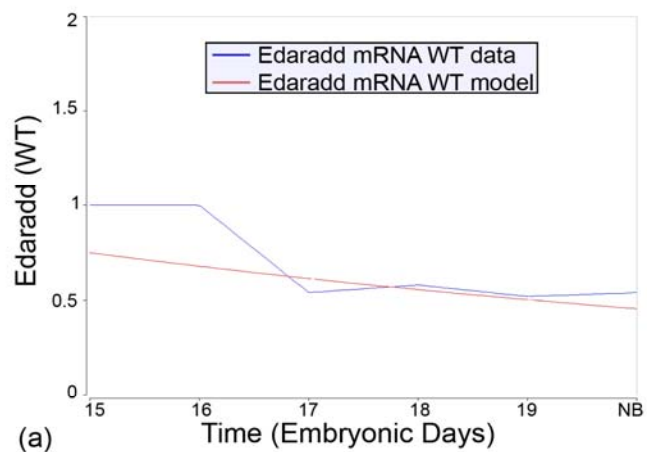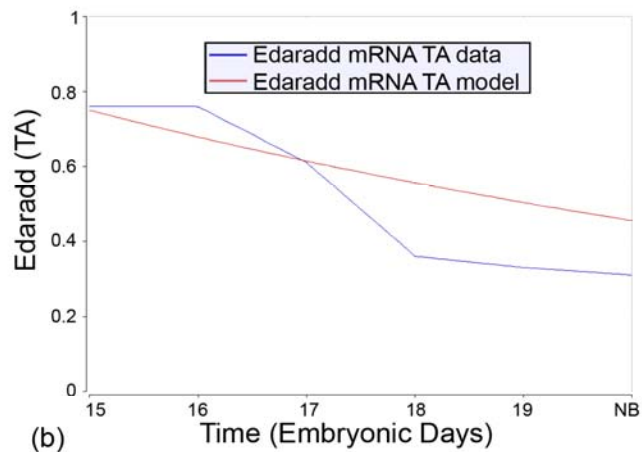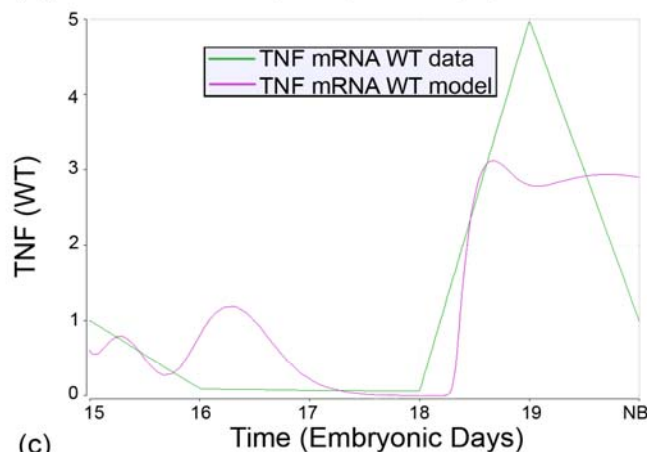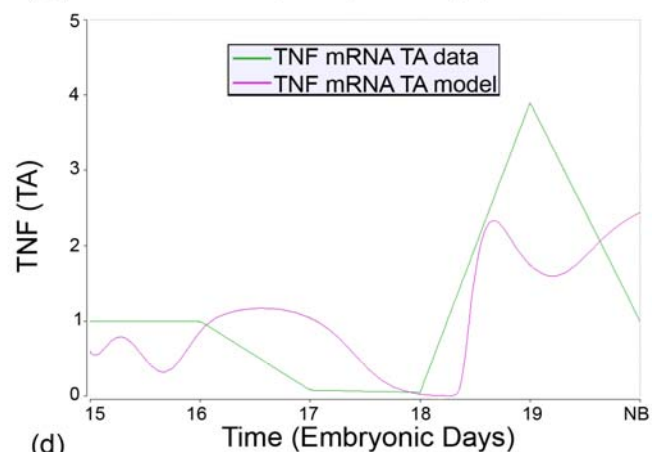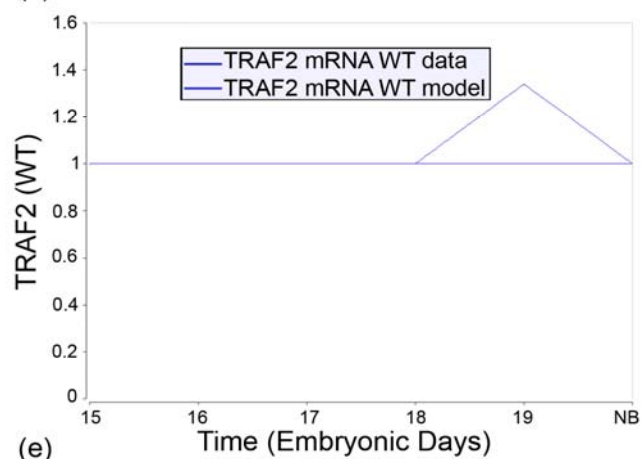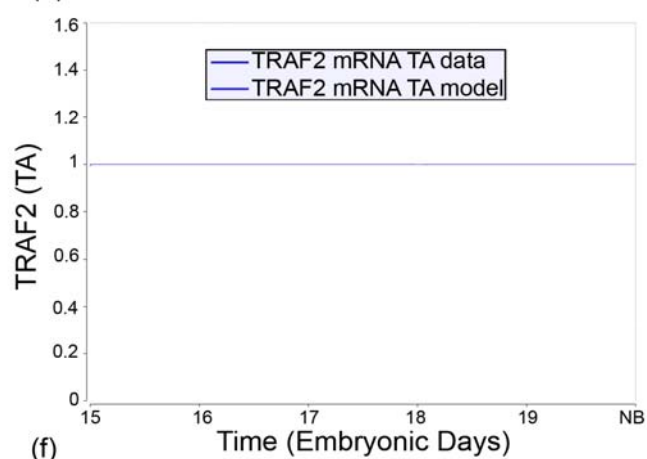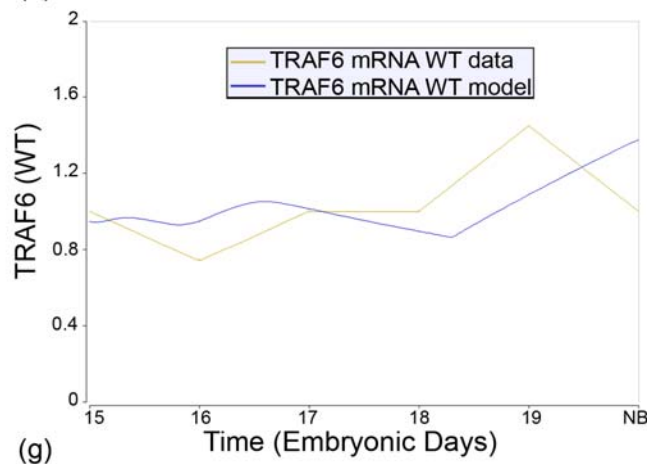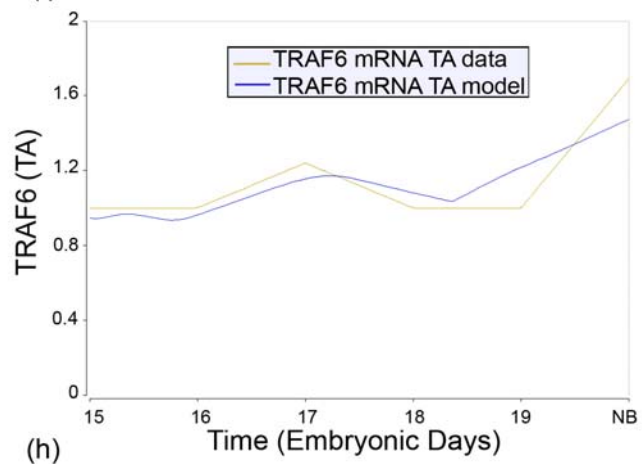

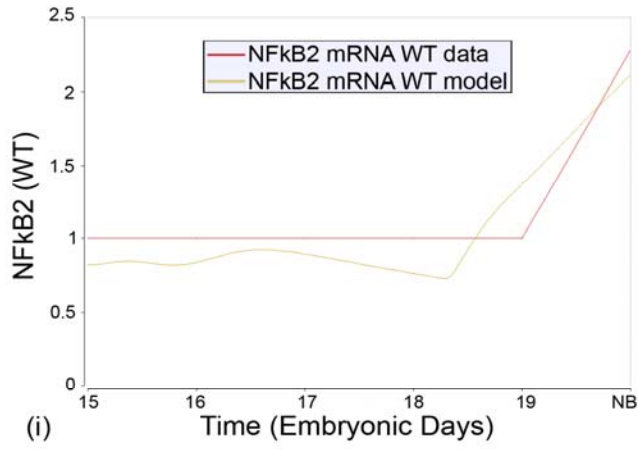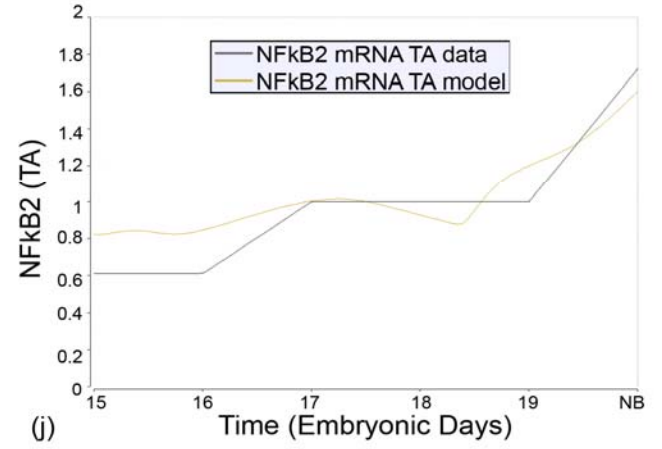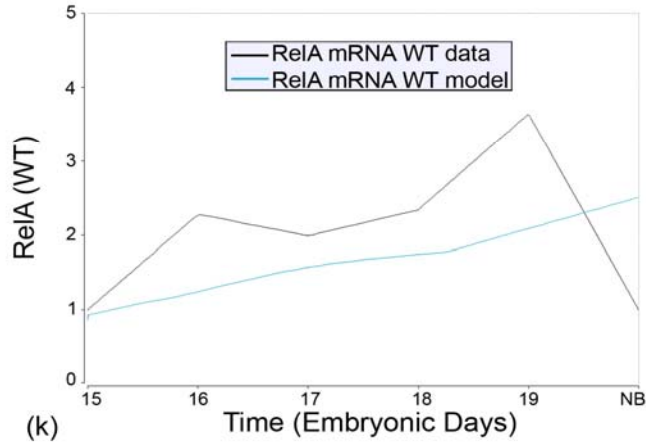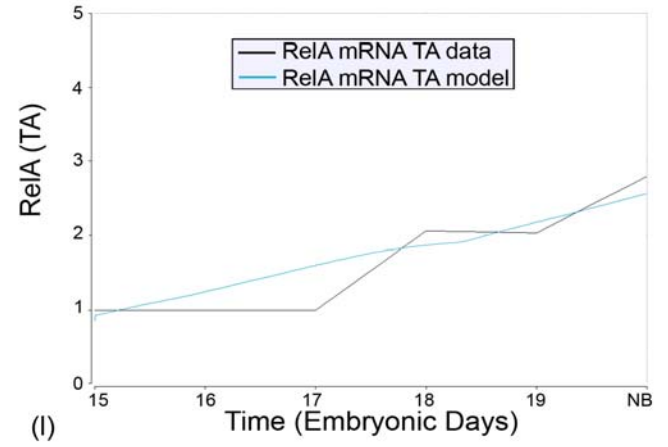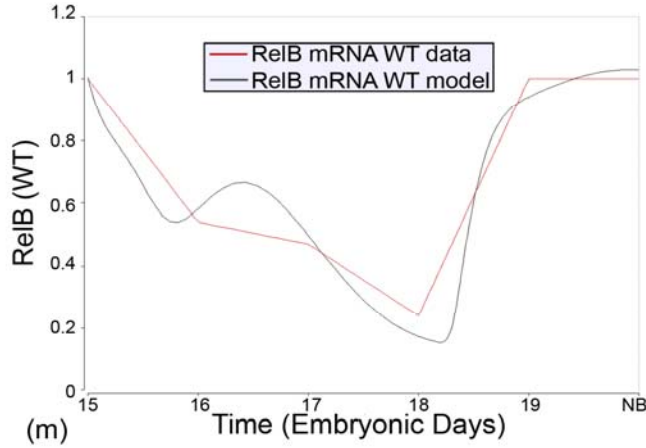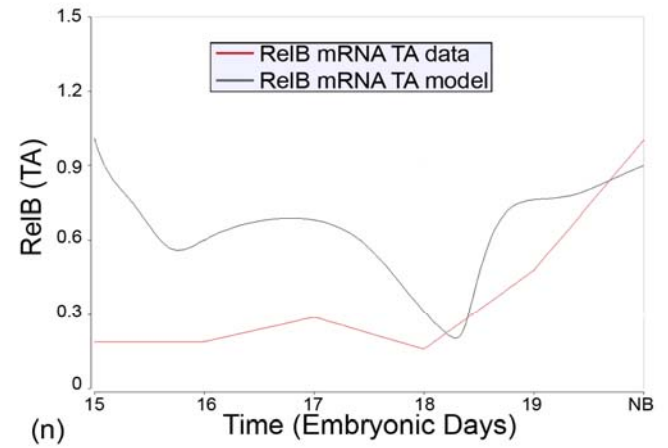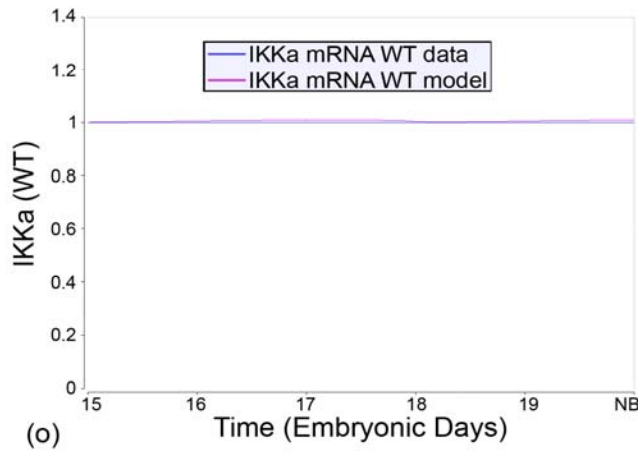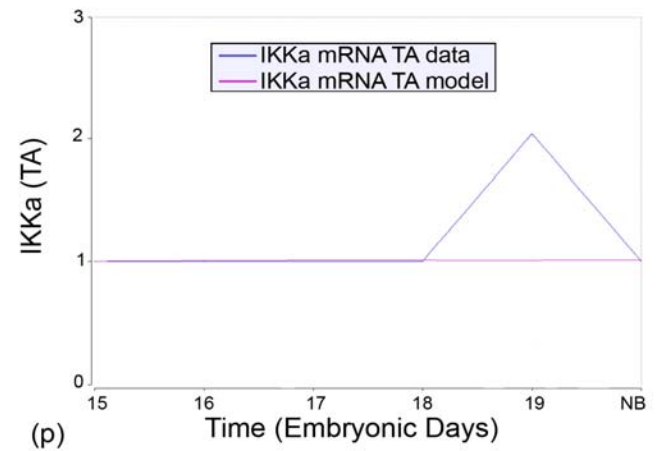

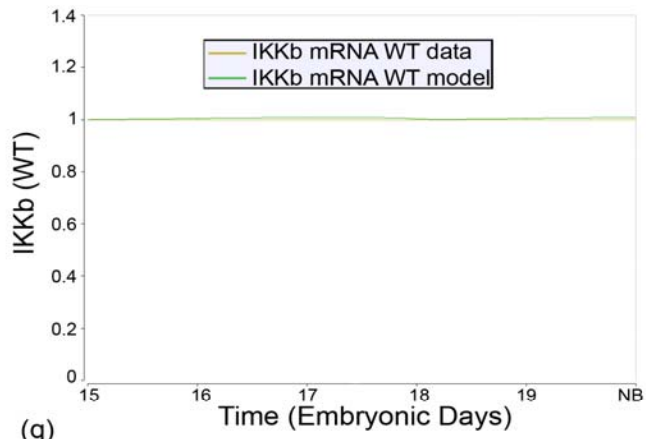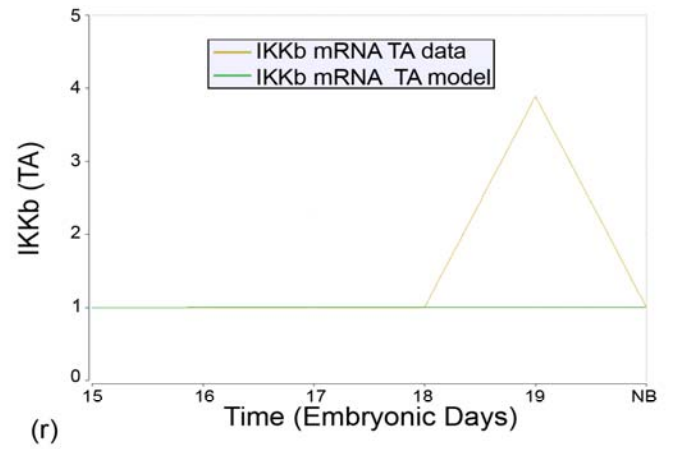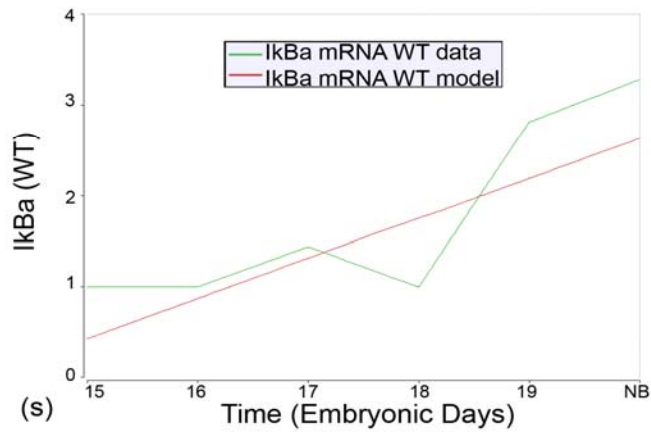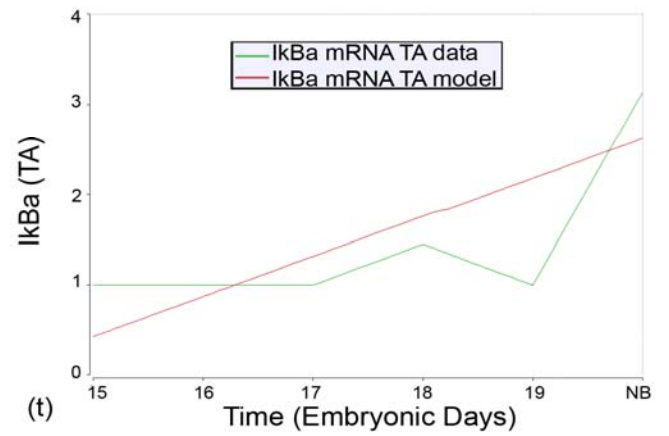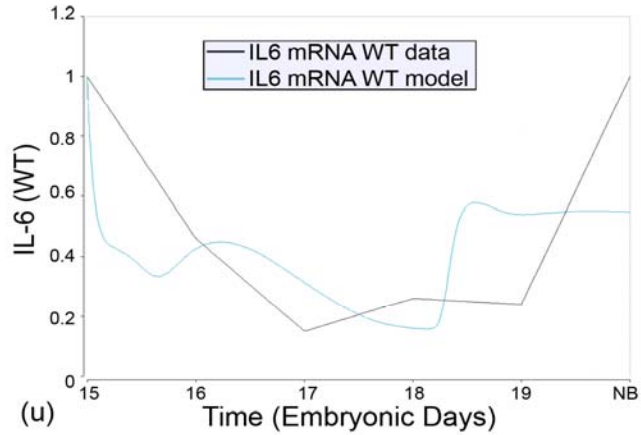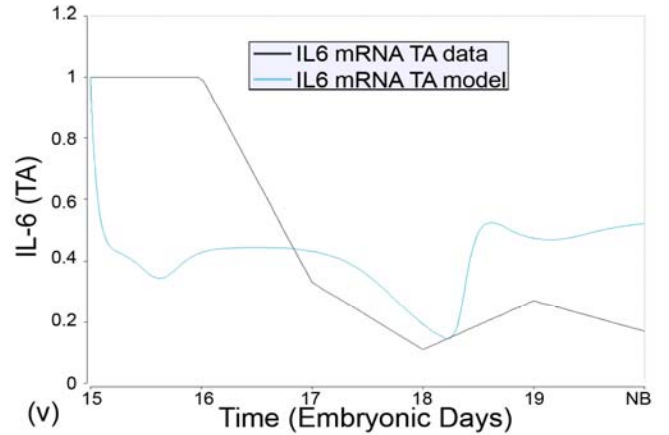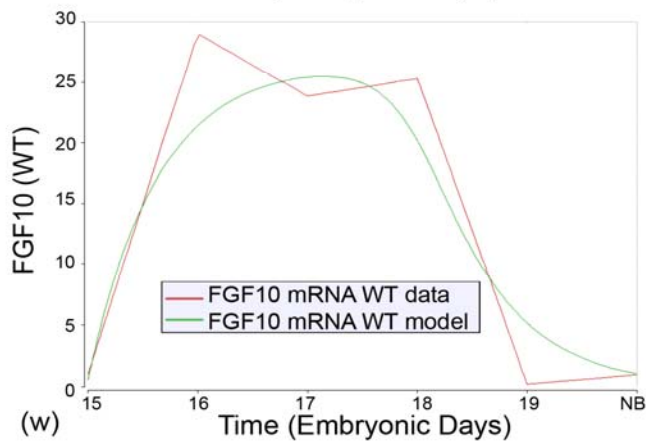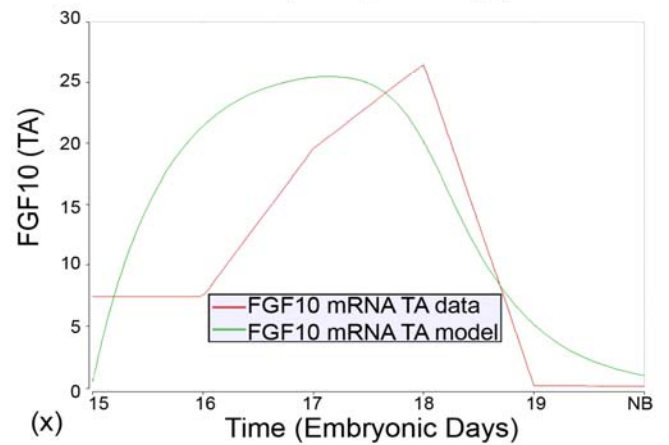

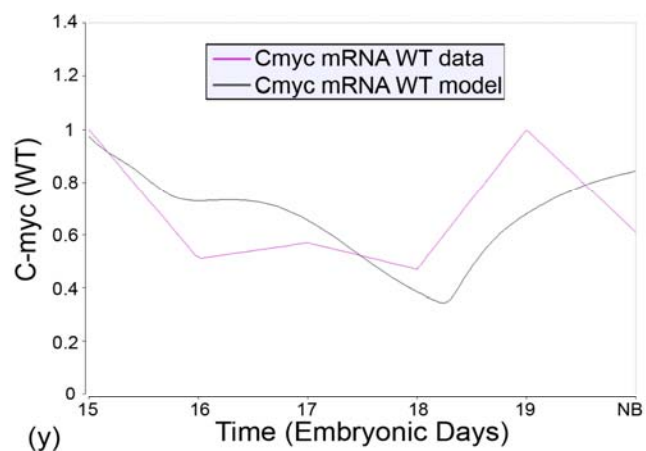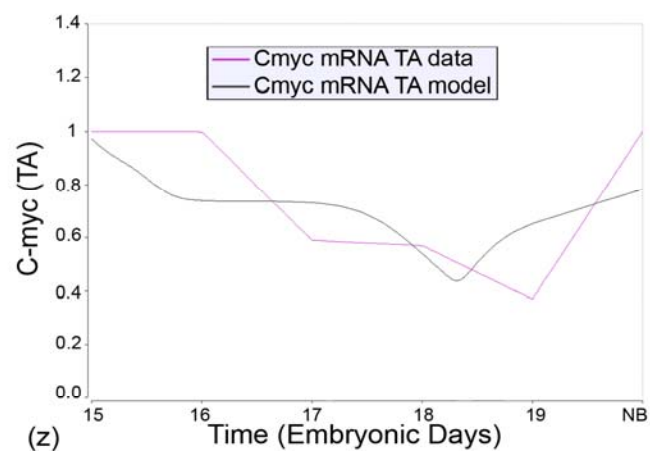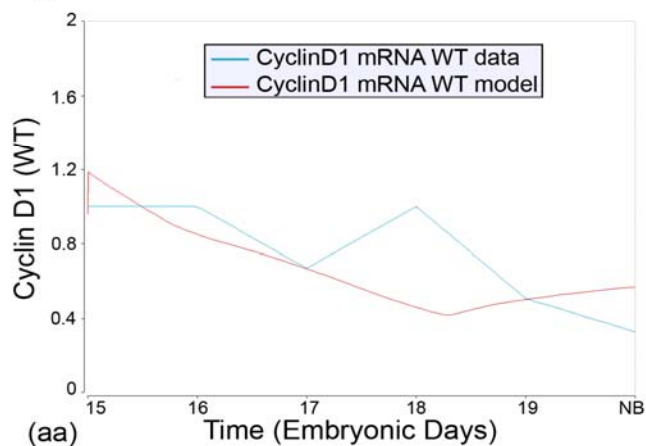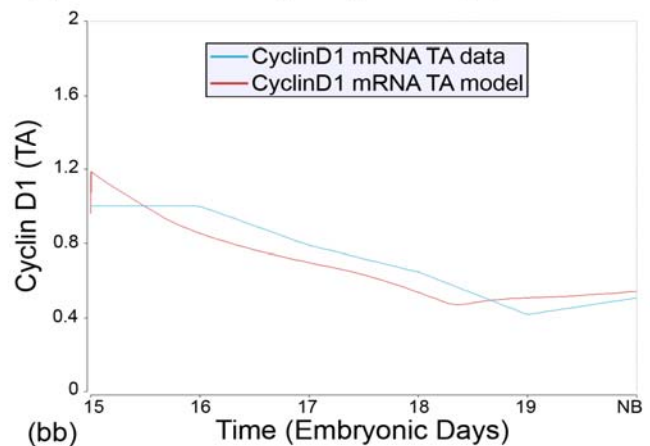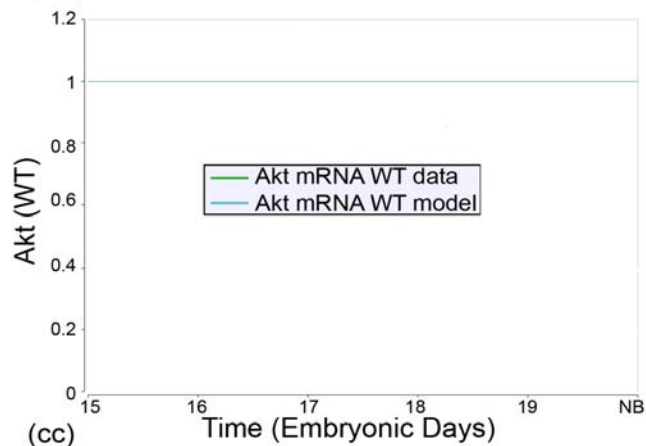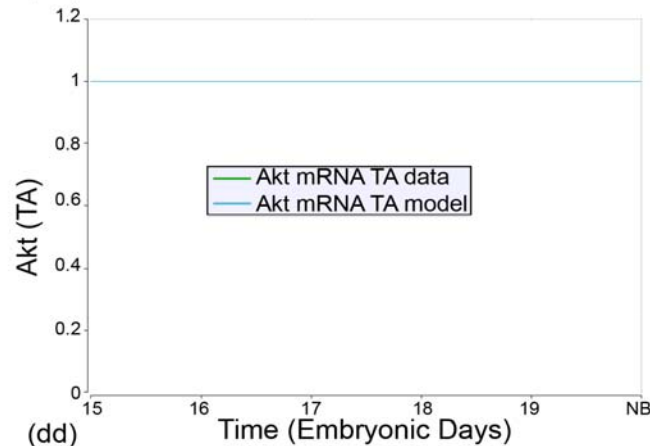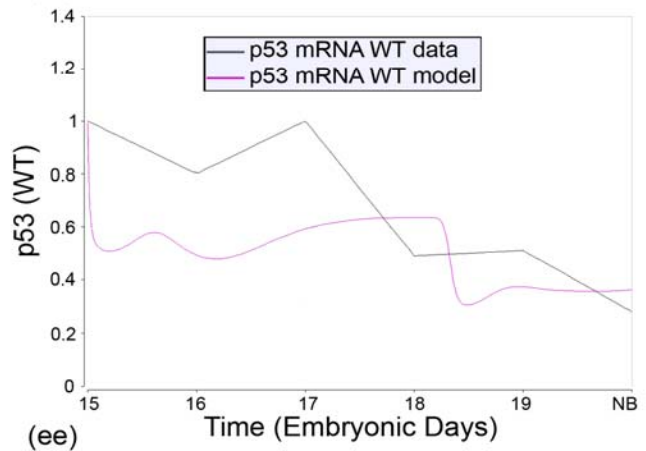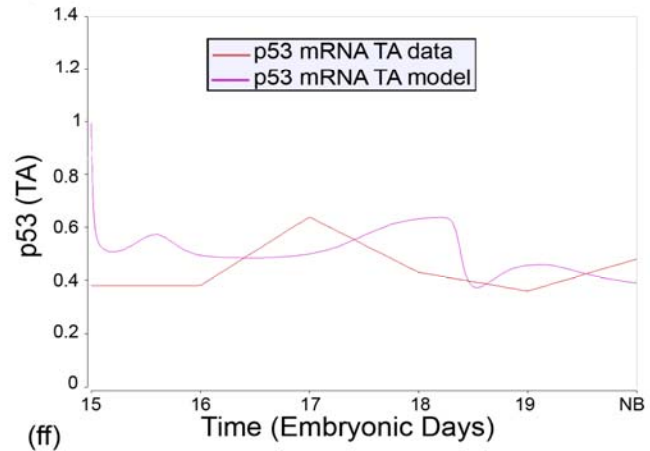

Supplement: Additional file 2 — Additional time course fits of the WT and Tabby experimental data by the full mechanistic model. Time course fits of the WT and Tabby experimental data by the full mechanistic model that are not included in Figures 4, 5, 8 of the main text. Note, Eda is not included among the genes because differences in Eda expression in WT and Tabby mice are not attributable to transcriptional control. The vertical axis is the relative abundance of mRNA, as presented in Tables 1 and 2. The lines labeled "WT data" are the quantitative RT-PCR derived mRNA data in wildtype SMGs; the lines labeled "WT model" are model simulated expected mRNA expression for wildtype SMGs. The lines labeled "TA data" are the quantitative RT-PCR derived mRNA data in Tabby SMGs; the lines labeled "TA model" are model simulated expected mRNA expression for Tabby SMGs mice. [file 1471-213X-9-32-S2.pdf]
